# Eryptosis as a marker of Parkinson's disease

**DOI:** 10.18632/aging.100695

**Published:** 2014-10-30

**Authors:** Etheresia Pretorius, Albe C Swanepoel, Antoinette V Buys, Natasha Vermeulen, Wiebren Duim, Douglas B Kell

**Affiliations:** ^1^ Department of Physiology, Faculty of Health Sciences, University of Pretoria, Arcadia 0007, South Africa; ^2^ Microscopy and Microanalysis Unit, University of Pretoria, Arcadia 0007, South Africa; ^3^ Department of Neurology Faculty of Health Sciences, University of Pretoria, Arcadia 0007, South Africa; ^4^ School of Chemistry and The Manchester Institute of Biotechnology, The University of Manchester, Manchester M1 7DN, Lancs, UK

**Keywords:** Parkinson's disease, hypercoagulability, erythrocytes, eryptosis

## Abstract

A major trend in recent Parkinson's disease (PD) research is the investigation of biological markers that could help in identifying at-risk individuals or to track disease progression and response to therapies. Central to this is the knowledge that inflammation is a known hallmark of PD and of many other degenerative diseases. In the current work, we focus on inflammatory signalling in PD, using a systems approach that allows us to look at the disease in a more holistic way. We discuss cyclooxygenases, prostaglandins, thromboxanes and also iron in PD. These particular signalling molecules are involved in PD pathophysiology, but are also very important in an aberrant coagulation/hematology system. We present and discuss a hypothesis regarding the possible interaction of these aberrant signalling molecules implicated in PD, and suggest that these molecules may affect the erythrocytes of PD patients. This would be observable as changes in the morphology of the RBCs and of PD patients relative to healthy controls. We then show that the RBCs of PD patients are indeed rather dramatically deranged in their morphology, exhibiting eryptosis (a kind of programmed cell death). This morphological indicator may have useful diagnostic and prognostic significance.

## INTRODUCTION

### Background

Parkinson's disease (PD) is a chronic neurodegenerative disorder leading to progressive motor impairment, and affecting more than 1% of the over-65 population [1, 2]; after Alzheimer's disease it is the second most common age-related neurodegenerative disease [3-7]. The neuronal population associated with the motor symptoms of PD is represented specifically by the dopaminergic neurons [8] of the substantia nigra pars compacta [3, 8-10]. Its degeneration leads clinically to progressive motor deficits including bradykinesia, rigidity, resting tremor, and postural instability, as well as a variety of non-motor symptoms in the later stages of the disease [11-14].

Unfortunately, most cases of PD are idiopathic, since their causes are both highly heterogeneous and often unknown [4, 7, 15-19]; further, there is currently no available cure for PD [5, 14]. Genetic studies have shown roles for so-called *PARK* genes that have been implicated in the disease, especially in the development of the monogenic forms of PD [6, 10, 20, 21]. As well as the genetic component, there is also a great body of evidence that suggests that non-genetic factors may also contribute of the development of PD (e.g. [10, 22-27]. Indeed, it is probable that there is an intimate interplay between genetic and environmental factors, and that the products of *PARK* genes may be involved in the increased susceptibility of neurons to environmental factors, leading to oxidative stress and (apoptotic) cell death [4, 10, 28-31]. Associated pathophysiology that may be due to both genetic and non-genetic factors may include mitochondrial dysfunction inflammation [32], abrogation of the autosomal-lysosomal autophagy system [33] and endoplasmic reticulum stress [4, 34, 35].

**Figure 1:**
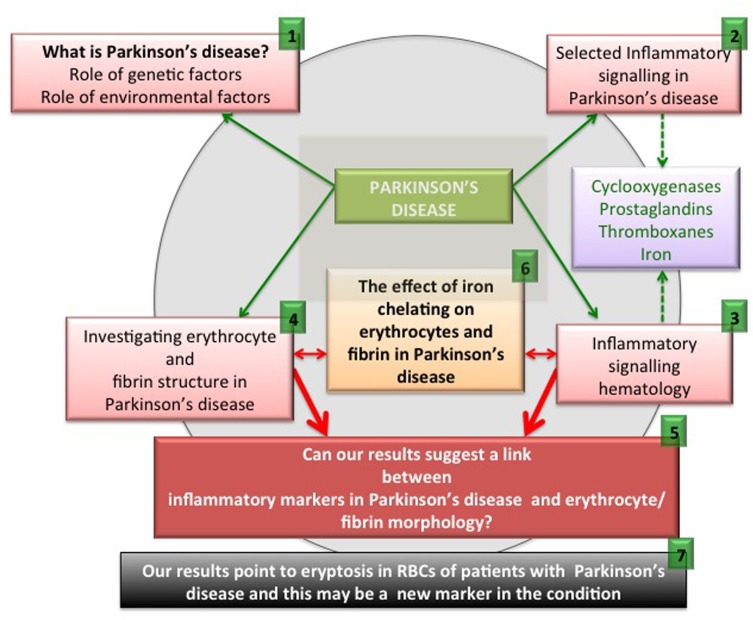
An overview figure summarizing the contents of this manuscript.

Unsurprisingly, no “one-step” approach alone explains the entire pathophysiology system of PD (e.g. [15, 16]), and therefore Funke and co-workers [10] and Balling and colleagues [36-38] have suggested that a systems biology approach is probably the most sensible way to look at the disease. A focus of in recent PD research is the investigation of biological markers that could help in identifying at-risk individuals or to track disease progression and response to therapies [1, 12], while (notwithstanding the differences between neuroinflammation and peripheral inflammation [39] inflammation is a known hallmark of PD and of many other degenerative diseases [33, 40-42]. In the current work, we therefore focus on inflammatory signalling in PD, using a systems approach that allows us to look at diseases in a more holistic way. Figure 1 shows an overview of the flow of the current manuscript.

As set down in Figure 1, we briefly discuss pathophysiology caused by genetic and environmental factors ((1) in the figure). We also look at selected inflammatory signalling molecules in PD, with a focus on cyclooxygenases, prostaglandins, thromboxanes and also iron (2). It is also well known that these particular signalling molecules that are involved in PD pathophysiology are also very important in an aberrant coagulation/hematology system (3). Therefore, we look at erythrocytes (RBCs) and fibrin networks from 30 PD patients to determine if their RBCs and fibrin networks are changed (4). Lastly, we present and discuss a hypothesis regarding the possible interaction of these aberrant signalling molecules implicated in PD, and suggest that these molecules may affect the coagulation/hematology system of the patients (5). To test this hypothesis further, we investigate the possibility that the iron chelator, desferal, might have an effect on RBCs and fibrin networks in PD (6). Finally, we show that eryptosis occurs in the RBCs of PD patients and that it may be an important marker of the disease (7).

The following paragraphs will look closely at the inflammatory signalling previously implicated in the progression of PD, with a focus on cyclooxygenases, prostaglandins, thromboxanes and iron. As usual, when we refer to ‘iron’ we do not normally specify either its valency or its speciation [43-45].

### Inflammatory signalling in Parkinson's disease

Despite the fact that the brain is an immune-privileged site, innate and adaptive immune responses do regularly take place in the brain [40]. While the interplay between inflammation and neuronal dysfunction is complex, there is evidence that chronic inflammation and innate immunity play prominent roles in PD [46], and that peripheral, as well as brain, inflammation contributes to the onset and progression of the neurodegenerative processes seen in PD [39, 47-51]. Thus, serum levels of tumor necrosis factor (TNF) are elevated in PD patients and the serum levels of interleukin-6 (IL-6) correlate with the Hoehn and Yahr staging [42, 46, 52, 53]. PD patients with dementia also showed significantly higher levels of C-reactive protein (CRP) compared to non-demented PD patients (p=0.032) and to the reference group (p=0.026) [54]. Also, CRP and fibrinogen values were found to be higher than in a control group [55].

**Figure 2:**
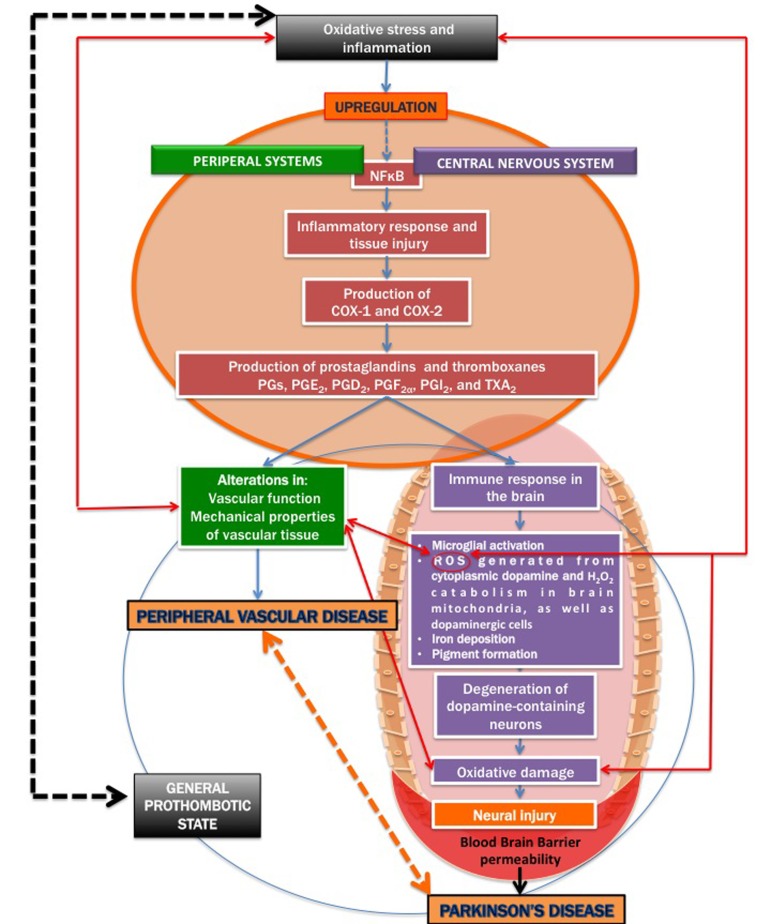
A simplified diagram on how inflammatory signalling contribu tes to both cardiovascular disease and Parkinson's disease.

There is also an increased blood brain barrier (BBB) permeability in PD patients [42, 56-58], as well as cerebral capillary dysfunction [59]. We therefore argue that in PD, a general upregulation of inflammatory signalling may affect both peripheral and brain tissue, resulting in a systemic inflammatory profile, mainly due to the resulting oxidative stress. Figure 2 shows a simpli-fied diagram of how inflammatory signalling can act to be a causal factor for both cardiovascular disease and PD.

It is known that severe oxidative stress and inflammation activate the redox-sensitive nuclear transcriptional factor kappa B (NFκB) (e.g. [60-66], resulting in an inflammatory response and tissue injury, including via the production of the cyclooxygenases cyclooxygenase-1 (COX-1) and cyclooxygenase-2 (COX-2) [67-69]. COX-1 and COX-2 are of similar molecular weight, approximately 70 and 72 kDa, and elevated production of prostanoids (the subclass of eicosanoids consisting of the prostaglandins, the thromboxanes, and the prostacyclins) from the constitutive COX-1 or inducible COX-2 is involved in the alterations in vascular function, structure and mechanical properties observed in cardiovascular diseases [68, 70-72].

Inflammation (whether peripheral or neuro-inflammation) may also lead to immune responses in the brain, COX-2 in particular, has also been implicated in neurodegenerative conditions [73-75]. Inducible COX-2 and the constitutively expressed COX-1 catalyze the first committed step in the synthesis of PGs, PGE_2_, PGD_2_, PGF_2α_, PGI_2_, and TXA_2_ [68, 76, 77]. COX-mediated neuronal injury is thought to be due to downstream effects of one or more prostaglandin (PG) products, including PGE_2_, PGD_2_, PGF_2α_, PGI_2_ and thromboxanes (particularly TXA_2_) that effect cellular changes through activation of specific prostaglandin receptor subtypes and second messenger systems [69, 76, 78].

Prostaglandins are potent oxygenated lipid molecules that contribute significantly to physiologic and pathophysiologic responses, including in the brain [79]. More specifically, PGE_2_ plays a central role in brain diseases, including in ischaemic injury and in several neurodegenerative diseases [72, 80, 81]. Importantly, PGs are produced in the peripheral systems, but also in resident brain cells [82]. Microglia and astrocytes act as immune cells in the inflamed brain and they both contribute to the onset of inflammation in many brain diseases (including PD) by producing deleterious pro-inflammatory mediators [83-87]. PGs, which are critical mediators of inflammation [68, 72, 80, 88, 89], are largely produced by activated microglia and reactive astrocytes during brain inflammation [90]. However, circulating inflammatory molecules have the ability to target their cognate receptors at the level of the blood-brain barrier; the latter in return is said [91] to produce specific prostaglandins (PGs).

The PGs exercise their effects by activating rhodopsin-like transmembrane-spanning G protein-coupled receptors (GPCRs) [77]. In particular, PGE_2_, which has been implicated in PD [79], signals through a class of four E-prostanoid (EP) receptors (EP1–EP4) [68, 88]. It has also been shown that, in many neurodegenerative models, the PGE_2_ EP_2_ receptor mediates a significant neuro-inflammatory response [68, 89, 92, 93].

Together with the thromboxanes and prostacyclins, the prostaglandins form the prostanoid class of fatty acid derivatives, a subclass of eicosanoids [94]. The thromboxane, TXA_2_ has prothrombotic properties and is formed from PGH_2_ by platelets and causes platelet aggregation. An upregulation of thromboxanes (in particular, TXA_2_) has also been found in PD animal models [95, 96]. As mentioned previously, TXA_2_ is an eicosanoid and is a lipid signalling mediator [97].

The immune response in the brain leads to microglial activation, elaboration of pro-inflammatory cytokines, the production of reactive oxygen species (ROS) [98], iron deposition [99, 100]; pigment formation [101, 102], and also secondary neuronal injury [68]. Dopamine, via the oxidative stress that it generates in the cytoplasm, could contribute to the selective loss of neurons observed in PD; cytoplasmic dopamine and the resulting ROS therefore is an important damage-pathway in PD [103]. Mitochondrial ROS, particularly imperfect respiration producing H_2_O_2_ in brain mitochondria has been implicated in PD [104]. The inflammatory response in the CNS plays a critical role in the pathogenesis of PD [68]. Oxidative damage in particular, plays prominent roles in the degeneration of dopamine-containing neurons [41, 105, 106].

### The link between prostaglandins, thromboxanes and iron accumulation in Parkinson's disease

There is extensive evidence that an overload of unliganded iron plays an important role in PD and in related disease such as progressive supranuclear palsy (PSP) [10, 22, 43, 44, 99, 107-141]. Iron is especially high in the substantia nigra, and it is, in particular, the hydroxyl radical produced via Fenton chemistry of the reaction between poorly liganded ferrous iron and hydrogen peroxide that is the chief culprit [142]. There is also evidence for a role of specific iron-dopamine complexes in the aetiology and pathogenesis of PD [43, 44, 138].

There is evidence that serum ferritin levels in PD patient might not be increased [123, 143, 144]. However, in a 2012 study, SF levels were significantly increased in male and female PD patients, and were correlated with Yahr stage and PD duration in men and women [143]. It was also shown that, in PD patients, there might be an accumulation of iron in the brain, which may accelerate free radical formation, lipid peroxidation, and neuronal death, but a reduction in systemic serum iron [145].

The question now arises as to whether increased iron (systemic or in the brain) is associated with the upregulation of COX-2, PGs and thromboxanes. Interestingly, already in 1990, it was suggested that iron overload causes an upregulation of PGs [146]. Literature also suggests a positive correlation between increased SF and prostaglandin PGF_2α_ [147], albeit SF is itself probably an inflammatory biomarker in many cases [45]. Iron overload also enhances the production of arachidonic acid and prostanoid, resulting in an increase in the production of TXA_2_ [148, 149] and prostacyclin [94].

The above-mentioned inflammatory signalling molecules are not only involved in PD, but are also well known for their participation in aberrant behavior of the coagulation/hematology system. The next paragraphs will discuss their effects specifically on the erythrocytes (RBCs) and on the fibrin network involved in clot formation. In the previous paragraphs, we reviewed the signalling molecules, cyclooxygenases, prostaglandins, thromboxane and iron, and showed their involvement and upregulation in PD. These molecules are also prominently involved in the hematological/ coagulation system. Here we focus specifically on platelets and fibrin, involved in the coagulation cascade, as well as RBCs (for recent reviews see [45, 150-153]. During inflammation, fibrin clot formation is changed [154-157] and this is due to a upregulation in signaling molecules – this will be discussed in the following paragraphs.

Cyclooxygenases (both COX-1 and COX-2) are expressed in platelets and have been widely studied from the perspective of the pharmacological properties of aspirin [158-162]. COX-1-dependent and -independent mechanisms also enhance Platelet-RBC interactions [163]. As mentioned in the previous paragraphs, cyclooxygenases mediate the production of PGs and thromboxanes. Both these signalling molecule groups are also found in platelets. Notably, the TXA_2_ receptor and PGI_2_ receptor are found in platelets [164]. Furthermore, PGs are also found in RBCs, these cells express prostacyclin receptor (PGI2) and contain PDE5 [165].

Thromboxanes (particularly TXA_2_) are produced by activated platelets and have prothrombotic properties and stimulate activation of platelets and increases platelet aggregation [166-169]. This is achieved by inducing expression of the glycoprotein complex GP IIb/IIIa in the cell membrane of platelets [164, 170, 171]. In platelets, TXA_2_ is generated from prostaglandin H2 by thromboxane-A synthase [172]. Also, the elevation of platelet reactivity by RBCs is likely mediated in part by its capacity to increase platelet TXA_2_ synthesis, and RBCs modulate biochemical and functional responsiveness of activated platelets [172, 173].

**Figure 3:**
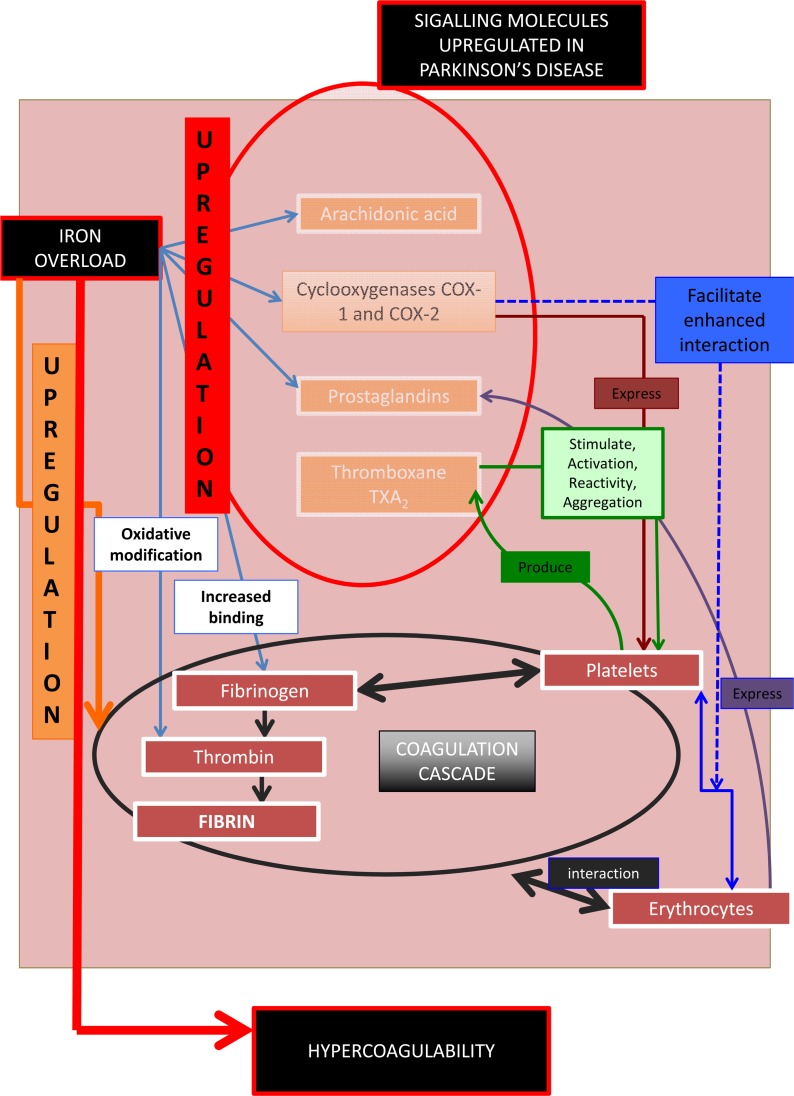
Signalling molecules, their role in the coagulation and hematological system, and their involvement in Parkinson's disease.

We summarize the above in Figure 3. Increased serum iron (mirrored in serum ferritin [45]) upregulates signalling molecules involved in the hematological/ coagulation system [174, 175]. This causes increased activation, stimulation and reactivity of platelets and a generally enhanced prothrombotic activity, resulting in hypercoagulability. These signalling molecules, including iron, are themselves unregulated in PD, suggesting that there may be a general hypercoagulable state in PD. Part of the purpose of the present work was to test this hypothesis.

Previously (see Table 1) we have shown a changed RBC and fibrin ultrastructure in various inflammatory diseases, in both humans and in animal studies. RBCs lose their shape and become elongated, while fibrin fibre networks become matted and more dense.

**Table 1:** Some diseases and hypercoagulable states in human and animal models associated with a changed erythrocyte and/or fibrin network structure

Hypercoagulable state	Cells/structures with ultrastrastructural changes	Reference
Alzheimer's Disease	RBCs and fibrin	[45, 176]
Diabetes	RBCs and fibrin	[45, 153, 157, 177-179]
Dysfibrinogenemia	Fibrin	[180]
HIV/AIDS	Platelets	[181, 182]
Hereditary hemochromatosis		[154, 183]
Hypercoagulability due to smoking	RBCs and fibrin	[156, 184, 185]
Lupus and Rheumatology	RBCs and fibrin	[45, 186-188]
Murine model: Asthma	Platelets and fibrin	[189, 190]
Pro-thrombin mutation	Platelets, RBCs and Fibrin	[191]
Rabbit model: aspartame	Platelets and fibrin	[192]
		
Rat model: ischemic stroke:	RBCs and fibrin	[193, 194]
Thrombo-ischemic stroke	RBCs and fibrin	[195-201]

Increases in iron levels play a major role in these conditions. Iron upregulation (in the sense of the release of poorly liganded iron) causes changes in both RBC and fibrin morphology [45, 153, 176, 183]. We have recently shown that added iron may impact on the coagulation profile and RBC ultrastructure [197, 202]. We have also documented that ferric ions can activate non-enzymatic blood coagulation, resulting in the formation of fibrin-like dense matted deposits (DMDs) observable by SEM [155, 203, 204]. Azizova and co-workers also noted that iron causes oxidative modification of thrombin [205]. RBCs also change their morphology under elevated iron levels [206]. All of these changes can be ascribed to the presence of iron, whether by electrostatic means directly, or through its role in the production of hydroxyl radicals (HR) [207-210].

In summary, Figure 3 shows that the prostanoid signalling molecules discussed have effects in both PD and on the haematology/coagulation system. One of the important pathophysiological actions of this upregulation in the haematology/coagulation system is a general state of hypercoagulability. This involves a changed coagulation system due in particular to the effects of thromboxanes on platelets reactivity and increased aggregation, ultimately causing pathologic fibrin formation. The up-regulation of the cyclo-oxygenases, prostaglandins, as well as an increased iron level, result in a general inflammatory profile in both the PD and in the hematology/coagulation system. Therefore these signalling molecules change the coagulation profile to a general hypercoagulable state, well known in PD. The results of epidemiological studies of the relationship between PD and stroke have been conflicting. There is evidence that stroke is not more prevalent in PD patients [211, 212]. However, recently, evidence was presented that the prevalence of stroke in idiopathic Parkinson's disease patients (iPD) is higher than in the general population, and there is also a 2- and 4-fold increased risk of having stroke or transient ischemic attack (TIA) in iPD patients with diabetes and hyperlipidemia, respectively [213, 214].

As the presence of these signalling molecules affects the hematology/coagulation system, we now look closely at one of the most prominent cells in this system, namely the RBC, as it might be an important indicator of inflammation.

### Cell death of RBCs: are signalling molecules that are up-regulated in Parkinson's disease possibly implicated in eryptosis?

The signalling molecules discussed in the previous paragraphs and implicated in PD are closely involved in the development of the variant programmed cell death, known as eryptosis, that applies to RBCs [215-219]. Although a PubMed search indicates that eryptosis has not yet been reported in PD, the next paragraphs will argue (and we later show) that eryptosis may indeed be present in PD, due to the changed signalling molecule profiles, and mostly also because it is known that these molecules are also involved in a changed coagulation profile.

**Figure 4:**
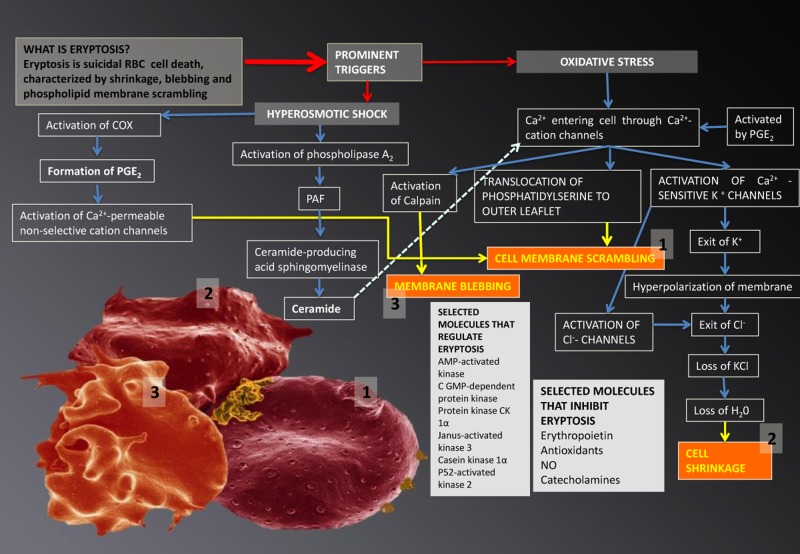
Eryptosis and its signalling molecules: the changes it effects on erythrocytes. Prominent triggers are hyperosmotic shock and oxidative stress. These triggers result in three morphologically visible changes to RBCs and these are phospholipid membrane scrambling (1); cell shrinkage (2) and membrane blebbing (3); when any of these changes are noted in RBCs, the phenomenon is collectively known as eryptosis.

Eryptosis is the suicidal cell death of mature RBCs, prior to hemolysis, and is characterized by cell shrinkage, blebbing [216, 218, 219] and membrane scrambling (phosphatidylserine translocation to the outer leaflet of the cell membrane with phosphate-dylserine exposure at the erythrocyte surface) [220]. Triggers of this process include oxidative stress, hyperosmotic shock, hyperthermia, as well as energy depletion [216-219, 221]. The signalling molecules reviewed in this article are involved here and act as triggers that mostly lead to an increased cytoplasmic Ca^2+^ [222], due to its entry through activated Ca^2+^ permeable channels. The following processes may happen in RBCs [215-219] (see Figure 4):

- Ca^2+^ may cause phosphatidylserine translocation [223] to the outer leaflet of the membrane with phosphatidylserine exposure at the erythrocyte surface [220]. This results in cell membrane phospholipid scrambling [217].
- Activation of Ca^2+^-sensitive K^+^ channels, causing the efflux of K^+^, resulting in a hyperpolarization of the RBC membrane. During this process, Cl^−^ leaves the cell, to assist charge balancing. This loss of KCl results in osmotic water flow, ultimately resulting in cell shrinkage.
- Activation of COX-2 followed by PGE_2_ production, causing activation of Ca^2+^-permeable non-selective cation channels, causing phospholipid membrane scrambling [217].
- Entry of Ca^2+^ followed by the activation of calpain, a cysteine endopeptidase that degrades membrane proteins, leading to membrane blebbing [218].
- Ca^2+^ sensitivity is enhanced by ceramide, which is another important player in eryptosis. Eryptosis is triggered by hyperosmotic shock, where activation of phospholipase A_2_ takes place, and this causes platelet activating factor (PAF) release (PAF is formed from cell membrane lipids, by phospholipase). Stimulation of the ceramide-producing enzyme sphingomyelinase, follows this. Ceramide increases the Ca^2+^sensitivity of the cell, resulting in phospholipid membrane scrambling.

### Signalling molecules: eryptosis in Parkinson's disease?

Research suggests that the above-mentioned signaling molecules in eryptosis are also involved in PD. The two signalling molecules that form a critical part in eryptosis, are calpain and ceramide, and the production of both these molecules has been found to be changed in PD [10, 224]. Two recent articles suggest that the calpains play a crucial role in the pathogenesis of PD [225, 226], and calpains are a family of calcium-dependent cysteine proteases that are ubiquitous in mammals and play critical roles in neuronal death [227].

Neurotoxins and alterations in Ca^2+^ homeostasis have also been associated with Parkinson's disease [228, 229]. In neurodegenerative disorders, cellular Ca^2+-^regulating systems are compromised, resulting in synaptic dysfunction, impaired plasticity and neuronal degeneration. Alterations of Ca^2+-^regulating proteins in the plasma membrane (ligand- and voltage-gated Ca^2+^ channels, ion-motive ATPases, and glucose and glutamate transporters), ER (presenilin-1, Herp, and ryanodine and inositol triphosphate receptors), and mitochondria (electron transport chain proteins, Bcl-2 family members, and uncoupling proteins) are implicated in neurodegenerative diseases [229]. Apoptotic cell death is well-known in PD, and here the endoplasmic reticulum (ER) and the mitochondria are in close communication, establishing a dynamic ER-Ca^2+^-mitochondria interconnection that can play a prominent role in the neuronal cell death seen in PD pathology [230].

Inhibition of eryptosis may occur mainly due to nitric oxide (which makes the RBCs resistant against increased cytoplasmic Ca^2+^), and erythropoietin (which inhibits Ca^2+^-permeable cation channels) [216, 231]. NO disrupts at least in part the effect of Ca^2+^ on cell phospholipid membrane scrambling [218] and by nitrosylation of enzymes [217]. Accelerated eryptosis is compensated by enhanced erythropoiesis and reticulocytosis [219]. Diseases associated with increased eryptosis (and that is also known for impaired microcirculation as well as a general inflammatory state) include diabetes, sickle-cell anemia, β-thalassemia, glucose-6-phospate dehydrogenase deficiency, malaria, iron deficiency, sepsis, renal insufficiency and hereditary spherocytosis [136, 217-219, 232]. During impaired microcirculation, eryptotic RBCs adhere to the vascular wall, by a mechanism involving phosphatidylserine at the RBC surface and CXCL16, as well as CD36 at the endothelial cell membrane [223]. Patients with PD are also known to have impaired microcirculation [233].

Given all the above, it follows that the possible interaction of these aberrant signalling molecules implicated in PD may affect the coagulation/hematology system, including RBC structure of PD patients (Figure 1(5)). However, there seems very little information available on the functioning of erythrocytes (RBCs) and the coagulation system in patients with PD. Here we therefore investigated the RBCs and fibrin fibre interactions of PD patients by using various microscopy techniques. Given the known and extensive involvement of iron in PD, we also look at the possibility that the iron chelator desferal might have an effect on RBCs and fibrin networks in PD (Figure 1(6)). Iron chelation possibly may protect substantia neurons in animal models of PD [234, 235]; as well as in PD patients [4, 139, 236-241]. In PD, there is an induction of transferrin receptor 2 expression and accumulation of oxidized transferrin inside mitochondria, which results in the release of labile ferrous iron from transferrin and the generation of hydroxyl radicals via Fenton chemistry [4, 242, 243]. Recently, it was suggested [4] that, in the case of PD, there is a need for agents able to appropriately “relocate” iron while at the same time limiting its participation in radical-generating reactions [244]. The iron chelator, de-feriprone, might have such an iron relocating ability [4].

## MATERIALS AND METHODS

### Parkinson's patient information

In the current manuscript, 30 healthy and 30 PD patients were studied. The healthy individuals did not smoke, nor use any chronic medication. The 30 PD patients were identified by a Neurologist and the diagnosis of these patients included the use of the Unified Parkinson's Disease Rating Scale (UPDRS). The Hoehn and Yahr scale (see Table 2) was used by the Neurologist to rate the relative level of the PD disability. Margaret M. Hoehn and Melvin D Yahr developed the Hoehn and Yahr scale to scale practically the severity of PD at the time of treatment, and thereby determine whether the medication or treatment that is used influences the rate of the progression of the disease [245]. Many studies thereafter have used this method in scaling the severity of movement disorders [246-250].

**Table 2:** Relative level of disability and stage of PA: the Hoehn and Yahr scale

Stage	Symptoms
Stage 0	No signs of disease
Stage 1	Symptoms on one side only (unilateral)
Stage 1.5	Symptoms unilateral and also involving the neck and spine
Stage 2	Symptoms on both sides (bilateral) but no impairment of balance
Stage 2.5	Mild bilateral symptoms with recovery when the ‘pull’ test is given (the doctor stands behind the person and asks them to maintain their balance when pulled backwards)
Stage 3	Balance impairment. Mild to moderate disease. Physically independent
Stage 4	Severe disability, but still able to walk or stand unassisted
Stage 5	Needing a wheelchair or bedridden unless assisted

Ethical clearance was obtained from the Health Sciences Ethical Committee of the University of Pretoria and informed consent was obtained from each of the patients as well as from controls (ethical number: 80/2013 under N Vermeulen and E Pretorius). Healthy individuals (individuals who did not smoke, use any medication or have any chronic diseases) also filled in consent forms. Two tubes of blood were obtained from each of these individuals (PD and controls): one 6mL citrate tube and the second 6 mL tube (iron or clotting tube), for serum ferritin (SF) analyses. The SF tests was done to determine whether the the PD patients have an increased SF level. Descriptive statistics for SF levels were calculated using MS-Excel; P values were calculated from the means, the numbers of objects measured in each class and the standard deviations using the Excel add-in available via http://www.talkstats.com/attachment.php?attachmentid=261&d=1213281245 and the facility at http://www.graphpad.com/quickcalcs/ttest1.cfm?Format=SD.

Whole blood (WB) was prepared with and without the addition of thrombin and also with and without the iron chelator desferal (final concentation of WB and desferal was 3.33 mM and WB with desferal and thrombin was 2.5 mM). Desferal was added to WB and left to react for 3 minutes. When thrombin is added an extensive fibrin network is created around trapped RBCs. Desferal was added to WB to determine whether there was a change in RBC shape and fibrin packaging density. These WB preparation methods were done for all microscopy techniques (described in the following paragraphs).

### Scanning electron microscopy (SEM)

High magnification SEM analyses were used to look at RBC structure and membrane surface. 10μL of the WB was dropped on a small glass coverslip to make smears with and without the addition of thrombin and desferal (as discussed in previous paragraphs) After the blood was collected, 10 μl of WB with and without 5 μl desferal were placed directly on a glass cover slip, fixed, dehydrated, dried, mounted and coated with carbon according to previously described methods [177]. Also, 10 μl of WB and 5 μl of desferal were mixed with 5 μl of thrombin, to create an extensive fibrin network in between the RBCs. The final concentation of desferal in WB was 3.33 mM and 2.5 mM, when thrombin and WB were also included with desferal. A Zeiss ULTRA Plus FEG-SEM with InLens capabilities was used to study the surface morphology of erythrocytes, and micrographs were taken at 1kV.

### Light microscopy (LM)

LM was used to study the axial ratios of erythrocytes (RBCs), using 100x magnification. 10ul of whole blood (WB) (with and without desferal) was used to make a thin smear on a microscopic glass slide, this smear was left for 24 hours to air dry followed by fixing for 5 minutes in 100% methanol and left to air dry for 30 minutes. The smears were stained again for 4 minutes with methylene blue, and rinsed under running water followed by air-drying for 30 minutes. The final staining step involved staining for 30 seconds in eosin and rinsing with running water. Slides were viewed using a Nikon Optiphod transmitted light microscope.

Axial ratios were determined from the LM micrographs, with the use of a program written in the C# programming language. The longest axis from each RBC was determined, referred to as the major axis, after which a perpendicular line was drawn in the centre of the major axis to establish the minor axis length. The axial ratio for each cell was obtained by dividing the major axis length by the minor axis length; a value of 1 represents a perfect circle.

### Atomic force microscopy (AFM)

Preparation was done according to previously described methods [176]. Characterization of RBCs with and without the addition of desferal was performed with a commercial AFM system (Dimension Icon with ScanAsyst, Bruker, USA) using the PeakForce QNM (Quantitative Nano-mechanical Property Mapping) imaging mode [251]. At every pixel point a rapid force-distance curve is performed and as the cantilever's deflection sensitivity and spring constant is calibrated before measurements, the curve can be analysed quantitatively to obtain a series of specific property maps of the sample. A retract curve is used to calculate modulus and adhesion images [153, 176, 252], the variation between the zero and maximum force is used to calculate deformation and the area between the approach and retract curve can be used to calculate energy dissipation [253]. Thus, the retract curve is used to calculate modulus and adhesion images (slope of the curve and the minimum of the curve respectively), the variation between the zero and maximum force is used to calculate deformation. The Young's modulus (a measure of the stiffness of an elastic material) was obtained from the slope of the force distance curve of the image using Derjaguin–Muller–Toporov (DMT) Model [153, 254]. Silicon Nitride probes (TAP525 – MPP 13120-10, Bruker, USA) with a nominal force constant of 200 N.m^−1^, a resonant frequency between 430 and 516 kHz (measured by the manufacturer), and a nominal tip radius of 15 nm were employed in all AFM measurements.

Ten randomly selected cells from each sample were analysed by selecting a 1μm by 1μm scan area on the periphery of the RBC and performing 128 by 128 data points of individual force curve measurements with a peak force of 6 μN. This is an extremely time-consuming analysis and can take up to 8 hours to analyse 10 cells. The periphery of the cells was chosen as there might be differences in concavity of RBCs, and we therefore chose an area that is not affected by the concavity of the specific RBC. The scans were performed at 0.6 Hz, which translates to a tip velocity of 1.2 μm/s and 25 to 35 force curves were chosen randomly within the stated area. Offline software (NanoScope Analysis version R3, Bruker, USA) was used to process the force curves and fit the modulus model to the unloading portion. The goodness of fit (R^2^) between the modulus model and the data given by the acquired curve is determined by calculating the ratio of explained variation to total variation in the dataset; only force curves with a goodness of fit so defined of 0.85 and above were used for modulus measurements.

The statistical significance of the difference between calculations was determined using one-way analysis of variance. A P value of less than 0.05 relative to the null hypothesis was considered to be ‘significant’ (cf. [255]), and P values are noted.

## RESULTS

Table 3 and Table 4 show the demographic information of the PD patients and healthy individuals, as well as their serum ferritin (SF) levels (healthy SF levels for males: 25 – 300 ng.mL-1 and females 25 to 200 ng.mL-1). Table 5 shows the medication profile of the patients, while Table 6 shows the medications used by the PD sample and a review of the literature to determine if the medication itself could possibly have an effect on RBC structure.

**Table 3:** Parkinson's disease patient (PD) demographic information

Patient ID	Age	Gender	Serum Ferritin (SF) values (ng.mL^−1^)	Hoehn and Yahr scale	Number of years with PD	Figure Number
**PD within normal serum ferritin ranges (normal levels for males: 25 – 300 ng.mL^−1^ and females 25 to 200 ng.mL^−1^)**						
3	61	Male	21	1.5	4	Figure 6: (H)
13	64	Male	21	1	6	Figure 7: (C) Figure 8: (D) Figure 10: (E;F)
18	76	Female	36	4	4	Figure 9: (C; D)
14	64	Female	60	3	2	
26	71	Male	60	1.5	4	
22	65	Female	65	2.5	?	
5	65	Male	68	3	5	
17	78	Male	80	4	13	Figure 6: (A)
24	71	Female	90	4	5	Figure 7: (H) Figure 8: (C)
28	70	Male	90	4	9	Figure 6: (C; D) Figure 9: (E; F)
7	84	Male	94	5	7	
1	82	Male	97	2.5	7	
15	73	Female	107	1	4	Figure 7: (D) Figure 9: (A; B)
16	67	Male	110	3	6	
8	71	Male	118	5	20	Figure 7: (A) 8: (B)
6	67	Male	125	5	17	
4	64	Male	126	2.5	16	
19	72	Female	145	3	8	Figure 6: (E; G) Figure 7: (E) Figure 8: (F) Figure 9: (G; H)
21	66	Male	156	3	7	Figure 9: (I; J)
20	69	Male	171	3	8	
23	80	Male	183	4	8	
11	68	Male	194	1.5	5	Figure 6: (B) Figure 7: (B) Figure 8: (A)
25	79	Female	125	5	30	
9	82	Male	281	3	7	Figure 8: (E)
**PD with High SF**						
29	48	Female	152	3	2	Figure 10: (C; D)
2	74	Female	212	2.5	10	
27	71	Male	358	1	1	Figure 6: (F) Figure 7: (F) Figure 8: (H) Figure 9: (K; L)
12	71	Male	372	1	2	Figure 7: (G) Figure 8: (G)
10	71	Female	405	5	5	
30	61	Female	568	1.5	4	Figure 10: (G; H)

**Table 4:** Healthy individuals demographic information

No	Gender	Age	SF ng.mL^−1^ 20-250	Figure number
1	F	45	13	Figure 10A
2	F	28	24	
3	F	27	28	
4	M	24	73	
5	M	63	48	Figure 5A to C Figure 10B
6	M	22	50	
7	F	55	64	
8	M	18	29	
9	M	25	125	
10	M	34	104	
11	M	30	156	
12	M	33	135	
13	F	25	36	
14	F	40	21	
15	F	31	58	
16	F	56	101	
17	M	23	62	
18	M	30	209	
19	M	23	128	
20	M	23	116	
21	M	23	65	
22	M	23	28	
23	M	23	109	
24	M	23	115	
25	M	23	70	
26	M	20	83	
27	F	48	37	
28	F	23	14	
29	F	56	65	
30	M	58	208	

**Table 5:** Medication used for each Parkinson's disease patient

Patient ID	Requip	Comtan	Sinemet	Stalevo	Pexola	Madopar	Carbilev	Azilect
1			√					√
2			√					
3								√
4	√		√					√
5					√			
6			√		√			√
7			√					
8	√		√	√				
9			√					
10								
11		√	√				√	
12						√		
13								
14						√		
15			√					
16			√					
17				√	√			
18								
19								
20						√		
21			√				√	
22								
23			√		√			
24							√	
25			√		√			
26			√					√
27								
28		√						
29					√			√
30			√					√

**Table 6:** Composition of medication and possible association of medication use for Parkinson's disease with erythrocyte changes or damage

PARKINSON'S DISEASE MEDICATION	WHY IS IT USED?	SELECTED REFERENCES	DOES IT HAVE AN EFFECT ON CARDIOVASCULAR OR ERYTHROCYTE FUNCTION?
Ropinirole (Requip®)	Rotigotine, a non-ergolinic dopamine-receptor agonist, is currently approved as monotherapy in early idiopathic Parkinson's disease in moderate to severe idiopathic restless legs syndrome.	[256-260]	Electrocardiogram (ECG) abnormalities were found in 40-52% of patients [261] **NO literature available on effect of medication on RBCs and eryptosis.**
			
Comtan® (entacapone)	Entacapone is an inhibitor of catechol-O-methyltransferase (COMT), used in the treatment of Parkinson's disease as an adjunct to levodopa and carbidopa therapy. COMT degrades catecholamines such as dopamine. Levodopa, a precursor of catecholamines, is an important substrate of COMT. COMT inhibitors, like entacapone, save levodopa from COMT and prolong the action of levodopa. Entacapone is a widely used adjunct drug of levodopa therapy.	[262-266]	Entacapone was not associated with an increased risk of acute myocardial infarction, stroke, or death in elderly patients with PD [267]. Entacapone is generally well tolerated, and no significant adverse events are reported [265]. Entacopone induces a dose-dependent inhibition of COMT activity inerythrocytes and a significant decrease in the plasma levels of 3-O-methyldopa, indicating their effectiveness as COMT inhibitors [268]. Entacapone, inhibit dose dependently the COMT activity in erythrocyts [269-271]. **NO literature available on effect of medication or COMT inhibition on eryptosis.**
Pramipexole Pexola®	Pramipexole is a nonergot dopamine agonists.	[272-274]	**NO literature available on effect of medication on RBCs and eryptosis.**
Rasagiline Azilect®	Rasagiline is a selective and irreversible monoamine oxidase (MAO) B inhibitor, which is well tolerated, safe, improves motor symptoms, and prevents motor complications in PD. Monoamine oxidases are a family of enzymes that catalyze the oxidation of monoamines. and catalyzes the oxidative deamination of monoamines MAO-B is mostly found in blood platelets.	[275-277]	In 80% patients MAO activity was considerably increased on 3-5 day after stroke [278]. MAO inhibitors appear promising as iron-chelators property [279]. **NO literature available on effect of medication on RBCs and eryptosis.**
**COMBINATION DRUGS**			
Stalevo® (carbidopa, levodopa, and entacapone)	Stalevo is an anti-parkinsonian dopaminergic combination medication that contains carbidopa, levodopa, and entacapone for the treatment of Parkinson's disease. Carbidopa inhibits aromatic-L-amino-acid decarboxylase (DOPA Decarboxylase or DDC).	[280-282]	**NO literature available on effect of medication on RBCs and eryptosis.**
SINEMET® (carbidopa-levodopa)	Levodopa (L-dopa) is the most widely used agent for the symptomatic relief of Parkinson's disease. Levodopa is the naturally occurring precursor amino acid for dopamine and the main therapeutic agent for neurologic disorders due to dopamine depletion, such as Parkinson's disease. Levodopa is converted to dopamine via the action of a naturally occurring enzyme, DOPA decarboxylase.	[283-288]	There was no increase in protein-incorporated dopa in erythrocytes [287]. Oxidative activity and specific oxidative activity of ceruloplasmin in serum and the activity of superoxide dismutase (SOD1) were not changed in PD erythrocytes [289] **NO literature available on effect of medication on eryptosis.**
Madopar® (levodopa + benserazide)		[290, 291]	**NO literature available on effect of medication on RBCs and eryptosis.**
Carbilev® (carbidopa + levodopa)	Levodopa combined with carbidopa is still the most effective treatment for symptoms of Parkinson's disease.	[292-294]	**NO literature available on effect of medication on eryptosis.**
**Amantadine not used by this PD population, but included in this table as it is known for its effect on RBCs**	Amantadine is a weak antagonist of the NMDA type glutamate receptor, increases dopamine release, and blocks dopamine reuptake.	[284, 295, 296]	Amantadine (5.0 mM) immediately disordered the packing state of the outer lipid leaflet of membranes [297]. RBCs express N-methyl D-aspartate (NMDA) receptors on their surface, but activation or inhibition of NMDA receptors on RBCs has no influence on their deformability and aggregability [298]. Side effects of amantadine include anemia and exposure of erythrocytes to amantadine increased [Ca2+] and triggered annexin V binding, suggesting that it may cause apoptosis[299]. Amantadine causes fluidization of erythrocyte membranes and inhibits vesicle release [300].

### Serum ferritin (SF) levels

The pathology laboratory, AMPATH in Pretoria, determined the SF levels of the patients as well as healthy individuals. The SF reference ranges of healthy individuals are taken between 20 and 150 ng.mL-1 for females and 20 and 300 ng.mL-1 for males. Table 3 shows the values for the SF levels of the PD patients, while Table 4 shows the values for the healthy individuals. There were no significant differences between SF levels of healthy individuals and PD patients. This supports selected references that have shown that, systemic SF in PD patients might not be increased [123, 143-145].

### Scanning electron microscopy (SEM)

**Figure 5:**
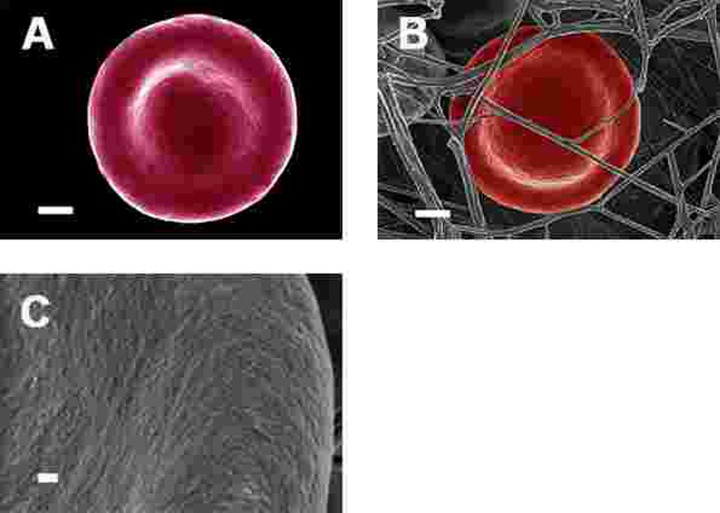
Representative erythrocytes of healthy individuals prepared from whole blood smears. Serum ferritin = 48 ng.mL^−1^ (**A**) Erythrocyte prepared from whole blood without thrombin; scale = 1μm; (**B**) Erythrocyte prepared from whole blood with added thrombin to create extensive fibrin fibre network around erythrocytes; scale = 1μm; (**C**) High machine magnification of 150,000x showing erythrocyte membrane, scale bar = 100 nm.

**Figure 6:**
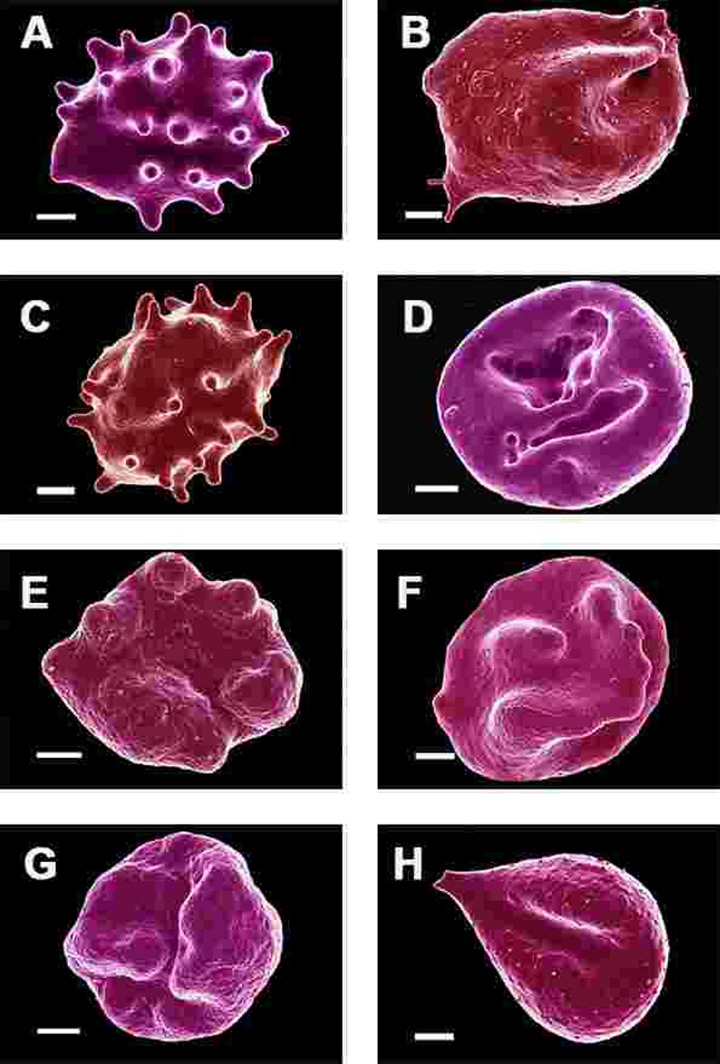
Erythrocytes of Parkinson's disease patients prepared from whole blood smears. Serum ferritin levels: (**A**) 80 ng.mL‐1 (**B**) 194 ng.mL‐1 (**C**) 90 ng.mL‐1 (**D**) 90 ng.mL^−1^ (**E**) 145 ng.mL‐1 (**F**) 358 ng.mL‐1 (**G**) 145 ng.mL‐1 (**H**) 21 ng.mL^−1^ Scale bar = 1μm.

**Figure 7:**
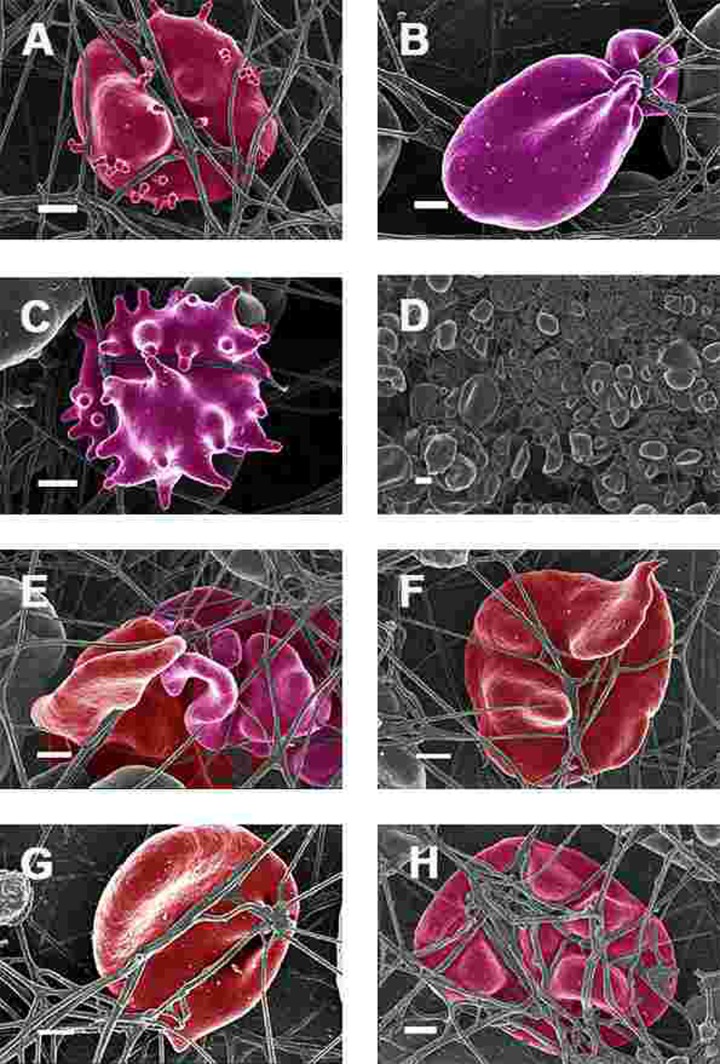
Erythrocytes of Parkinson's disease patients prepared from whole blood with added thrombin. Serum ferritin levels: (**A**) 118 ng.mL^−1^ (**B**) 194 ng.mL-1 (**C**) 21 ng.mL-1 (**D**) 107 ng.mL^−1^ (lower machine magnification to show general SEM view of erythrocytes) (E) 145 ng.mL^−1^ (**F**) 358 ng.mL-1 (**G**) 372 ng.mL-1 (**H**) 90 ng.mL-1 Scale bar = 1μm.

**Figure 8:**
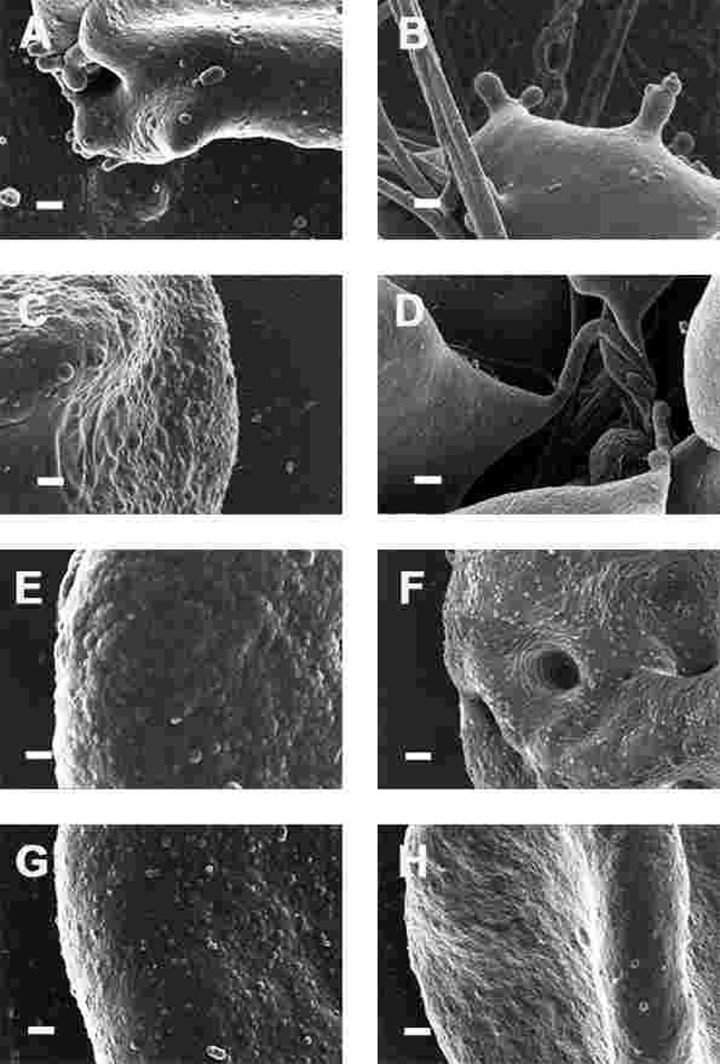
High magnification (100,000x machine magnification) of erythrocyte membranes of Parkinson's disease patients prepared from whole blood. Serum ferritin levels: (**A**) 194 ng.mL^−1^ (**B**) 118 ng.mL^−1^ (**C**) 90 ng.mL^−1^ (**D**) 21 ng.mL^−1^ (**E**) 281 ng.mL^−1^ (**F**) 145 ng.mL^−1^ (**G**) 372 ng.mL^−1^ (**H**) 358 ng.mL^−1^ Scale bar = 200 nm.

SEM micrographs of WB from both healthy individuals are shown in Figure 5 and PD patients are shown in Figure 6, 7 and Figure 8. Healthy RBCs dropped onto a glass cover slip show a typical discoid shape Figure 5A [45, 153, 176, 183, 191, 199, 301]. When thrombin is added to WB, an extensive fibrin network is formed over and around healthy RBCs, but without changes to the discoid RB C shape Figure 5B. High magnification of membranes, show a smooth membrane architecture Figure 5C. In the current study, we noted that most RBCs from PD patients do not have a typical discoid shape as seen in their axial ratios (see Figure 10C); instead they show typical eryptotic and/or elongated morphology. During eryptosis, the RBCs undergo phospholipid membrane scrambling, shrinkage and blebbing, blebbing that is particularly striking in the WB smears of the PD patients (Figure 6). We have shown previously (Table 1) that high iron levels that occur in various diseases may cause changes in RBC structure, where the cells become elongated (with limited prominent blebbing). This elongation was noted in a large fraction of the RBCs of high-SF Alzheimer's individuals (AD) [176], as well as in the hereditary iron overload disease, haemochromatosis (HH) and in individuals with high SF (HF individuals), but without the HH mutation [154]. In AD individuals with SF levels within the normal ranges, the RBC structure was not changed. In these high iron conditions (often correlated with high SF [45]), RBCs have an elongated, non-discoid RBC shape. These elongated shape changes are also induced when physiological levels of iron are added to healthy whole blood [199, 202, 204, 206].

In the current study, 24 of the 30 patients have SF values in the normal ranges. However, in all 30 individuals, including the patients with both normal and high SF levels, a changed (eryptotic) RBC structure was noted in most of the cells (See Figure 6 and Figure 10C for RBCs of PD patients, with their SF levels indicated in the legend).

When thrombin was added to WB, RBCs of PD patients folded and became entrapped in the fibrin fibre net (see Figure 7). In the patients with SF in the normal ranges, no changes fibrin fibre networks were noted, and were very similar to the fibrin nets of the controls (see Figure 5). While the numbers of cells shown in the SEM analyses are limited, similar findings were observed in both SEM and LM analysis. To give an indication of a typical SEM view on low magnification, Figure 7D shows the typical morphology of many RBCs entrapped in the fibrin.

This trapping was also previously noted in individuals with high SF diagnosed with Alzheimer's disease, hereditary hemochromatosis and hyperferritinaemia individuals [154, 176]. However, in PD, irrespective of SF levels, RBCs were non-discoid and folded around fibrin fibres (Figure 7). Figure 8 shows high magnification micrographs of the RBC membranes from a series of PD patients. Membrane ultrastructure appears much more rough and textured than that of healthy RBCs (in Figure 5C). We suggest that this is due to membrane phospholipid scrambling, associated with eryptosis [216-219, 302]. Here also, phospholipid membrane scrambling occurs in both high and normal SF level PD patients (Figure 8).

**Figure 9:**
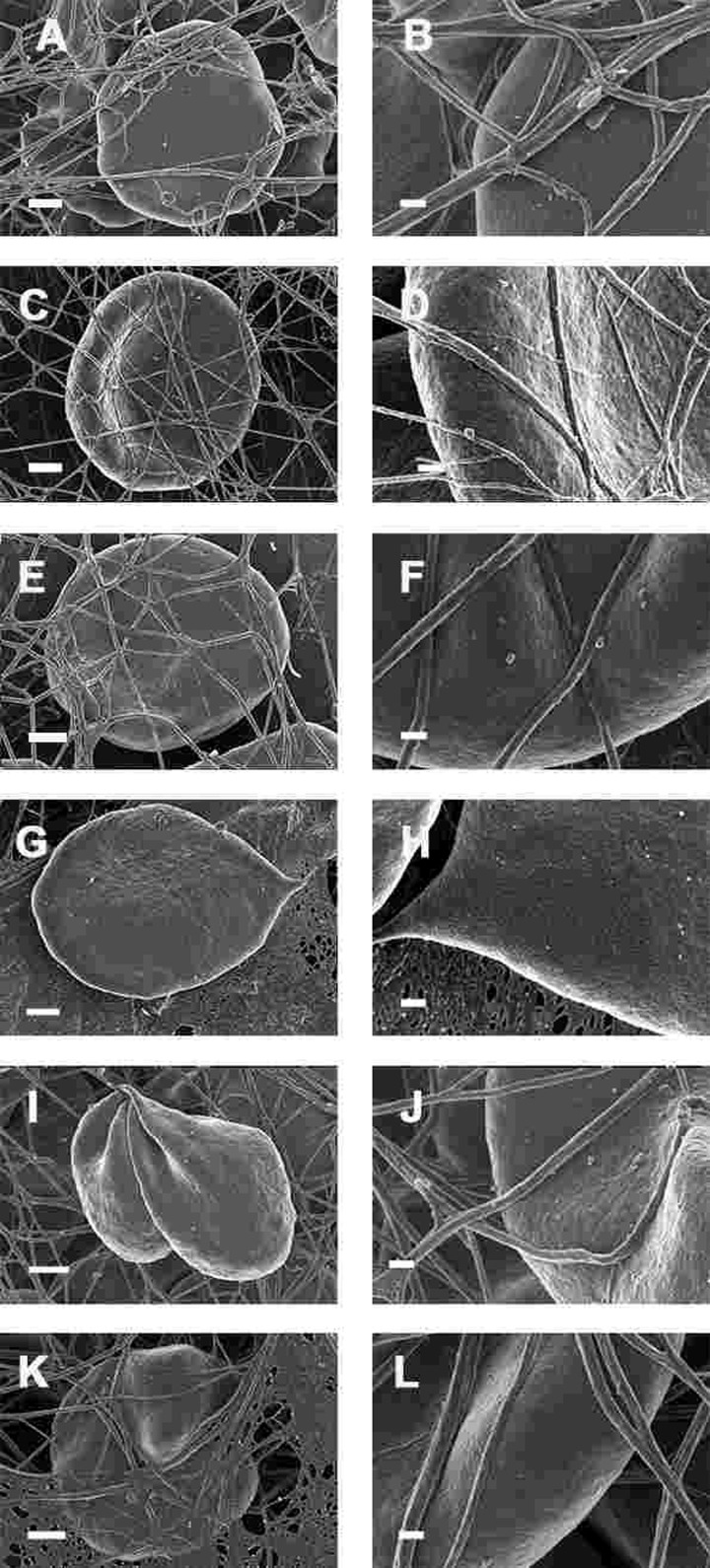
Whole blood of Parkinson's disease patients treated with desferal with added thrombin. All micrographs in left column are low magnifications while the micrographs in the right columns are the respective 100,000x machine magnification of the same individual's erythrocyte membranes. Serum ferritin levels: (**A**) and (**B**) 107 ng.mL^−1^ (**C** and **D**) 36 ng.mL-1 (**E** and **F**) 90 ng.mL^−1^ (**G** and **H**) 145 ng.mL^−1^ (**I** and **J**) 156 ng.mL^−1^ (**K** and **L**) 358 ng.mL^−1^ Low magnification scale bar = 1μm; high magnification scale bar is 200 nm.

We also studied the effect of the iron chelator desferal on RBC shape and membrane structure. Desferal is the classical clinically approved iron chelator; however, it has poor gastrointestinal absorption and therefore has to be administered intravenously or subcutaneously [303-305]. WB from PD patients with added desferal are shown in Figure 9 (all micrographs in left column are low magnifications while the micrographs in the right columns are the respective 100,000x machine magnification of the same individual's erythrocyte membranes). Desferal is an effective, hexadentate iron chelator and we have previously shown that when added to WB of HH and HF individuals, or of samples treated with free iron, it facilitated a shape change, where the RBCs reverted back to a more discoid shape. Here, we also noted a change to a more discoid shape; however, the gross shape changes were not as prominent in PD patients as was previously noted in HH and HF individuals [154]. This said, a very interesting observation at 100,000x magnification was that the membranes did revert to a smoother topography, suggesting that the desferal did have a stabilizing effect on RBC structure.

**Figure 10:**
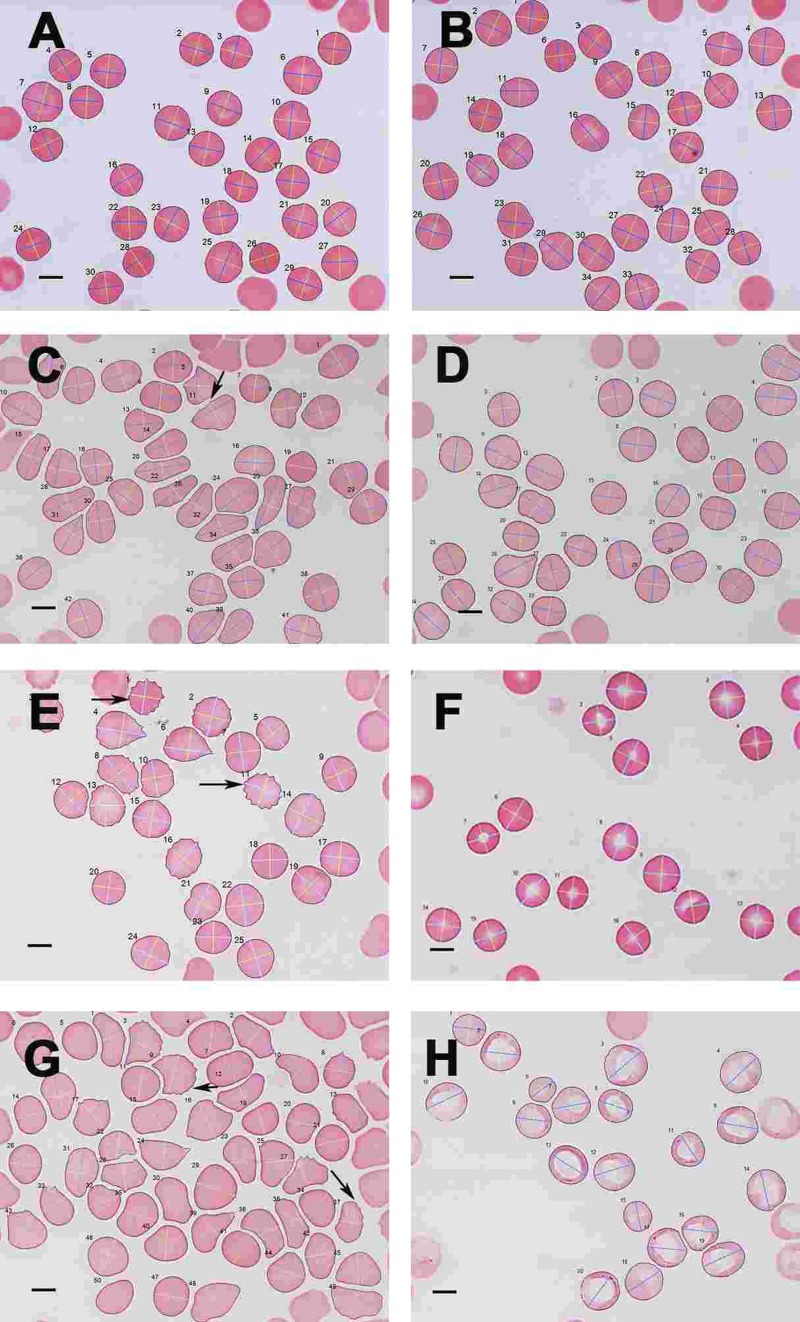
Light microscopy micrograph of whole blood smears from (**A**) a healthy individual (serum ferritin: 13 ng.mL-1) and (**B**) a healthy individual, (serum ferritin: 48 ng.mL^−1^) (**C**) a female PD individual (serum ferritin: 152 ng.mL^−1^) and (**D**) the same PD individual after treatment with desferal; (**E**) a male PD individual (serum ferritin: 21 ng.mL^−1^) and (**F**) the same PD individual after treatment with desferal; (**G**) a female PD individual (serum ferritin: 568 ng.mL^−1^) and (**H**) the same PD individual after treatment with desferal. Major and minor axes indicated on the RBCs, as determined by the C# program written for the analysis. Scale bar = 5 μm.

### Light microscopy (LM)

As SEM micrographs showed that most RBCs have a changed shape, micrographs of whole blood smears were taken using LM, to determine for much larger numbers of cells if there was a statistical difference in axial ratios between healthy RBCs and RBCs from PD patient Figure 10 C). Figure 10 A and B show a typical LM smear of a healthy individual with the major and minor axis as determined by the C# program, while Figure 10 C and D; E and F and G and H show micrographs from PF individuals before and after desferal treatment. In SEM analysis, we noted a definite change in some of the PD patients, when desferal was added to their whole blood; therefore axial ratios before and after desferal addition was also analyzed statistically. Table 7 shows the total number of cells analyzed for each group along with the group's average axial ratio and standard deviation. The p-values were determined with Student's t-test. A significant difference was found between the axial ratios of the RBCs of healthy individuals and those from both the PD and PD + desferal groups (p-values of 5.5 X 10^−6^ and 0.0006 respectively). No significant difference was found between PD and PD + desferal (p-value of 0.68). Previously we determined axial ratios of 20 cells per healthy individual, using a manual measuring method [154]. In this analysis the average axial ratio was 1.05. In the current analysis where many more cells were analysed per individual, the average healthy RBC axial ratio was 1.13.

**Table 7:** Average axial ratio and total number of cells analysed for healthy individuals, individuals with Parkinson's disease (PD) and these individuals treated with desferal

	Healthy individuals (N=30)	PD Individuals (N=30)	Blood from PD individuals treated with desferal (N=6)
**Total number of cells analysed**	2531	2409	1848
**Average**	1.13	1.18	1.17
**Standard deviation**	0.09	0.11	0.08

**Figure 11:**
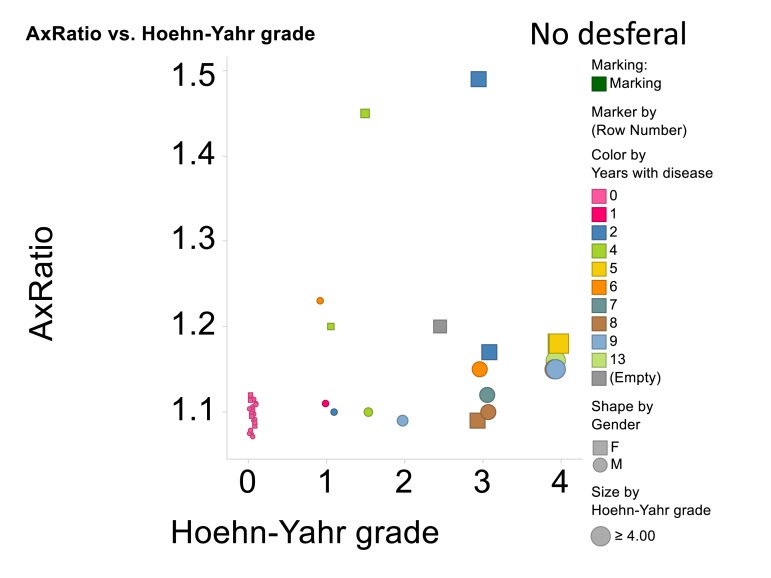
Relationship for all cases and controls between 3ratio and Hoehn-Yahr grade. No desferal was added. “Empty” means that these data on years with disease were not recorded.

A summary of all the light microscope data appears in Figure 11, that also encodes gender, time with disease and Hoehn-Yahr grade.

### Atomic force microscopy (AFM)

**Figure 12:**
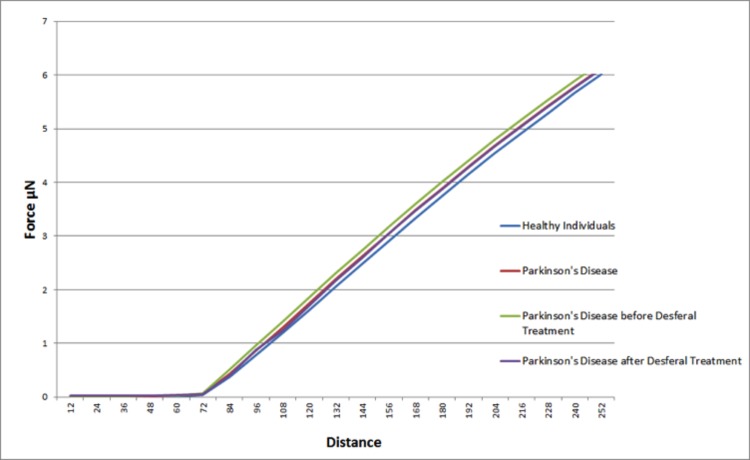
Representative force-distance curves obtained on erythrocytes from healthy individuals, individuals suffering from Parkinson's disease, individuals suffering from Parkinson's disease with high serum ferritin and the same individuals suffering from Parkinson's disease with high serum ferritin after treatment with desferal.

**Figure 13:**
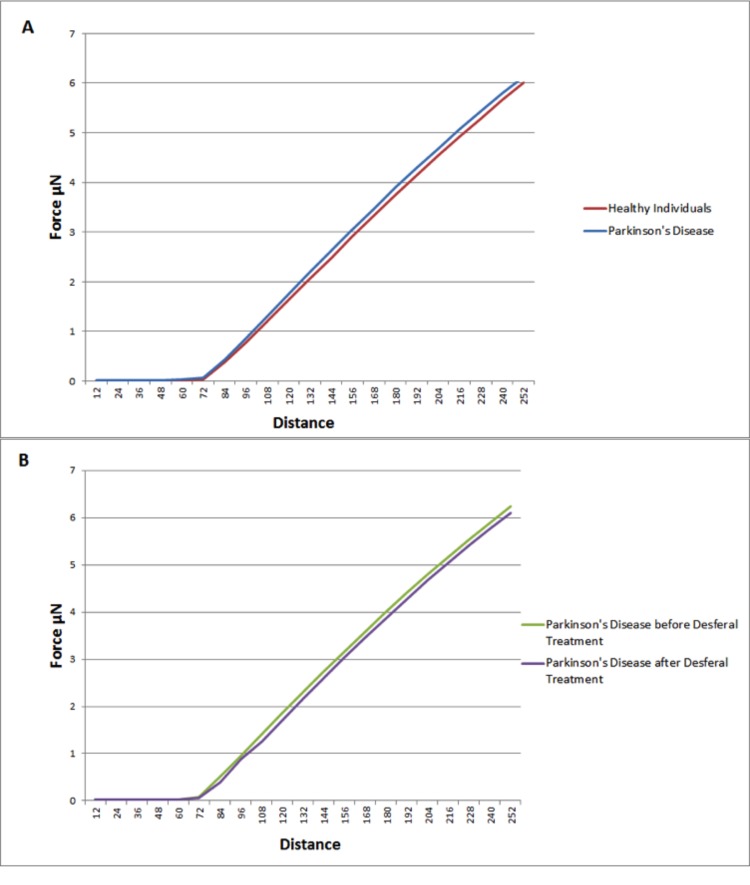
Representative force-distance curves showing the difference between: (**A**) Healthy individuals and individuals suffering from Parkinson's disease. (**B**) Individuals suffering from Parkinson's disease with high serum ferritin and the same individuals suffering from Parkinson's disease with high serum ferritin after treatment with Desferal. Force-Distance curves show the atomic force microscope (AFM) cantilever deflection range on the cell surface.

Elastic modulus calculations (Table 8) acquired from force-distance curves showed an increase in the Young's modulus of RBC's from patients suffering from Parkinson's disease when compared to RBC measurements from control subjects (51821 and 46711 MPa respectively); this increase of Young's modulus reflects a decrease in the elasticity of the cell membrane. A subset of the PD patients (6) showing elevated high iron levels were treated with the chelator desferal. Elasticity measurements before and after treatment showed a decrease in the Young's modulus of roughly 3000MPa after treatment (see Table 8), indicating a certain amount of recovery of cell membrane elasticity. These results can be visualized in force-distance curves (Figure 12), where a representative curve from an RBC from a patient with PD is compared to a curve from a healthy individual's RBC; a steeper slope can be seen for the healthy individual (Figure 13A) and a curve from a desferal-treated RBC (Figure 13B) shows a slightly flatter curve and increased displacement than a curve from an RBC not treated with desferal.

**Table 8:** Descriptive statistics for elasticity of red blood cell (RBC) membranes

	Mean	Standard Error	P value	N Individuals	N Cells	N Curves
**PARKINSON'S (PD)**	**51821**	846	0.0025	26	260	10376
**CONTROL**	**46710**	749		11	110	2737
						
**PD WITH HIGH SF BEFORE DESFERAL**	**56257**	1018	0.0457	6	60	3029
**PD WITH HIGH SF AFTER DESFERAL**	**53056**	1626		6	60	2073

## DISCUSSION

In the introductory paragraphs of this manuscript we pointed out that the signaling molecules associated with the presence of neuro-inflammation, seen in PD individuals are the same as those that are typically associated with cardiovascular inflammation [12, 33, 40, 42, 46, 69, 75, 88, 106, 306, 307] (see Figure 2 and Figure 3). We also argued that, when we look closer at eryptosis, the most common cell death mechanism of RBCs, the same signaling molecules implicated in PD is involved in eryptosis [215-219] (see Figure 4). Particularly, the two signalling molecules calpain and ceramide, that are prominently involved in eryptosis, are also implicated in PD [10, 224-226], and (particularly calpain) play critical roles in neuronal death [227].

This evidence that is presented from reviewing the literature, suggests that there is a case for looking closely at the RBC structure in PD individuals. Previously, it was shown that an increased red blood cell distribution width (RDW) is a strong predictor of mortality in the general population of adults 45 years or older [308]. Also, it was recently suggested that cytometric analysis of cell size and heterogeneity of size could be used as a potential biomarker [309].

In the current manuscript, we therefore looked at the ultrastructure of RBCs of PD patients, and noted that in whole blood smears, RBCs have a changed shape, and that most of the RBCs have a typical eryptotic shape (see Figure 6) and light microscopy smear of healthy individuals and PD individuals (see Figure 10 A to H). Axial ratios of healthy RBCs are 1.13 (see Table 7), where that of PG individuals have an average of 1.18. In the presence of thrombin (see Figure 7), RBCs are trapped in fibrin fibers that twist around the aberrantly-shaped RBCs. Very high SEM magnification (see Figure 8), showed that membranes of these RBC are much more granular than in those found in healthy RBCs (see Figure 5C). AFM analyses showed that there were significant differences in the membrane elasticity of PD individuals compared to that of healthy individuals. This confirms the possibility that phospholipid membrane scrambling is present in the PD RBC membranes.

In the current PD group the systemic SF levels were also mostly in the normal ranges, and although there were individuals with increased SF levels, we could not distinguish between the RBCs from normal versus high SF PD individuals. This differs from what we found in Alzheimer's individuals, where RBCs of patients with normal SF levels, looked similar to healthy individuals, but individuals with high SF levels had a changed ultrastructure and elasticity as seen with AFM [176]. There is therefore no specific relation between increased SF levels and aberrant RBC ultrastructure here, as all PD RBCs have a changed morphology (irrespective of SF level). This might be due to the major inflammatory nature of the condition. Previously, we showed that, when there is an increased systemic SF, such as in Alzheimer's disease and hereditary hemochromatosis, fibrin fibre morphology was also changed. The fibrin fibers formed thick and matted deposits and we suggested that it might be due to the oxidative stress as a result of the increased systemic SF or iron levels [176]. In the current sample, the fibrin fibres of the PD group with SF levels within the normal ranges did not show a denser fibrin fibre net. This might be due to the absence of increased systemic iron levels. We might therefore assume that the changed RBC shape seen in all the PD patients might be due to the upregulation of the inflammatory markers discussed in the previous paragraphs, and that the iron plays a less prominent role.

However, because of the established involvement of increased SF in PD [22, 111, 112, 116, 119, 122, 123, 129, 134-137], we looked at whether the iron chelator desferal might have an effect on the RBC structure of our PD sample. No statistically significant effect was noted. This might be taken to indicate that under the chronic conditions of PD the effects of iron are due more to Fenton chemistry than to electrostatics [45, 153, 154], and that up-regulated inflammatory markers may play a more prominent role in the changed RBC structure, than iron (SF).

We conclude by suggesting that PD should be treated not only as a neuro-inflammatory and neuro-degenerative condition because of the observable peripheral effects. There is striking evidence from the literature that suggests that PD patients might also have general cardiovascular inflammation [306, 307], and that this might lead to a general ill health of the patients. RBC morphology may be developed as markers that might reflect the severity and prognosis of PD in these patients. The heterogeneity of the RBC population appears to be a prognostic marker of aging/longevity and it is suggested that the RDW. Therefore, RDW may be another marker that may change with progression of PD. RDW is measured by flow cytometry on a population of 10,000 or more cells and therefore this use of this parameter should be investigated further. In particular, assessment of the RBC structure may be a simple diagnostic test for the impact of treatment regimes, and iron chelators may have a role to play in treating this very debilitating condition. Overall, we recognise that PD may have multiple causes and act via multiple pathways [10]. The fact that it can be mimicked, to some degree, by a variety of toxins [310], is consistent with the idea that there may be environmental triggers. We note too that iron chelation can prove effective in such models, too [237, 311-315].

## References

[1] Saracchi E, Fermi S, Brighina L (2014). Emerging candidate biomarkers for Parkinson's disease: a review. Aging and disease.

[2] Xu J, Gong DD, Man CF, Fan Y (2014). Parkinson's disease and risk of mortality: meta-analysis and systematic review. Acta neurologica Scandinavica.

[3] Reeve A, Simcox E, Turnbull D (2014). Ageing and Parkinson's disease: Why is advancing age the biggest risk factor?. Ageing research reviews.

[4] Isaya G (2014). Mitochondrial iron-sulfur cluster dysfunction in neurodegenerative disease. Frontiers in pharmacology.

[5] Romero-Ramos M, von Euler Chelpin M, Sanchez-Guajardo V (2014). Vaccination strategies for Parkinson disease: Induction of a swift attack or raising tolerance?. Human vaccines & immunotherapeutics.

[6] Berwick DC, Harvey K (2014). The regulation and deregulation of Wnt signaling by PARK genes in health and disease. Journal of molecular cell biology.

[7] Beitz JM (2014). Parkinson's disease: a review. Frontiers in bioscience (Scholar edition).

[8] Gibb WR (1992). Melanin, tyrosine hydroxylase, calbindin and substance P in the human midbrain and substantia nigra in relation to nigrostriatal projections and differential neuronal susceptibility in Parkinson's disease. Brain research.

[9] Seidl SE, Santiago JA, Bilyk H, Potashkin JA (2014). The emerging role of nutrition in Parkinson's disease. Frontiers in aging neuroscience.

[10] Funke C, Schneider SA, Berg D, Kell DB (2013). Genetics and iron in the systems biology of Parkinson's disease and some related disorders. Neurochemistry international.

[11] Maillet A, Pollak P, Debu B (2012). Imaging gait disorders in parkinsonism: a review. Journal of neurology, neurosurgery, and psychiatry.

[12] McGhee DJ, Royle PL, Thompson PA, Wright DE, Zajicek JP, Counsell CE (2013). A systematic review of biomarkers for disease progression in Parkinson's disease. BMC neurology.

[13] Palavra NC, Naismith SL, Lewis SJ (2013). Mild cognitive impairment in Parkinson's disease: a review of current concepts. Neurology research international.

[14] Pedrosa DJ, Timmermann L (2013). Review: management of Parkinson's disease. Neuropsychiatric disease and treatment.

[15] Berg D, Lang AE, Postuma RB, Maetzler W, Deuschl G, Gasser T, Siderowf A, Schapira AH, Oertel W, Obeso JA, Olanow CW, Poewe W, Stern M (2013). Changing the research criteria for the diagnosis of Parkinson's disease: obstacles and opportunities. Lancet neurology.

[16] Berg D, Postuma RB, Bloem B, Chan P, Dubois B, Gasser T, Goetz CG, Halliday GM, Hardy J, Lang AE, Litvan I, Marek K, Obeso J (2014). Time to redefine PD? Introductory statement of the MDS Task Force on the definition of Parkinson's disease. Movement disorders: official journal of the Movement Disorder Society.

[17] Gaenslen A, Wurster I, Brockmann K, Huber H, Godau J, Faust B, Lerche S, Eschweiler GW, Maetzler W, Berg D (2014). Prodromal features for Parkinson's disease - baseline data from the TREND study. European journal of neurology: the official journal of the European Federation of Neurological Societies.

[18] Gröger A, Bender B, Wurster I, Chadzynski GL, Klose U, Berg D (2013). Differentiation between idiopathic and atypical parkinsonian syndromes using three-dimensional magnetic resonance spectroscopic imaging. Journal of neurology, neurosurgery, and psychiatry.

[19] Lerche S, Seppi K, Behnke S, Liepelt-Scarfone I, Godau J, Mahlknecht P, Gaenslen A, Brockmann K, Srulijes K, Huber H, Wurster I, Stockner H, Kiechl S (2014). Risk factors and prodromal markers and the development of Parkinson's disease. Journal of neurology.

[20] Coppedè F (2012). Genetics and epigenetics of Parkinson's disease. TheScientificWorldJournal.

[21] Kilarski LL, Pearson JP, Newsway V, Majounie E, Knipe MD, Misbahuddin A, Chinnery PF, Burn DJ, Clarke CE, Marion MH, Lewthwaite AJ, Nicholl DJ, Wood NW (2012). Systematic review and UK-based study of PARK2 (parkin), PINK1, PARK7 (DJ-1) and LRRK2 in early-onset Parkinson's disease. Movement disorders: official journal of the Movement Disorder Society.

[22] Abergel RJ, Warner JA, Shuh DK, Raymond KN (2006). Enterobactin protonation and iron release: structural characterization of the salicylate coordination shift in ferric enterobactin. Journal of the American Chemical Society.

[23] Valko M, Leibfritz D, Moncol J, Cronin MT, Mazur M, Telser J (2007). Free radicals and antioxidants in normal physiological functions and human disease. The international journal of biochemistry & cell biology.

[24] Hosseini Tabatabaei N, Babakhani B, Hosseini Tabatabaei A, Vahabi Z, Soltanzadeh A (2013). Non-genetic factors associated with the risk of Parkinson's disease in Iranian patients. Functional neurology.

[25] de Lau LM, Breteler MM (2006). Epidemiology of Parkinson's disease. Lancet neurology.

[26] Allam MF, Del Castillo AS, Navajas RF (2005). Parkinson's disease risk factors: genetic, environmental, or both?. Neurological research.

[27] Di Monte DA (2003). The environment and Parkinson's disease: is the nigrostriatal system preferentially targeted by neurotoxins?. Lancet neurology.

[28] Irrcher I, Aleyasin H, Seifert EL, Hewitt SJ, Chhabra S, Phillips M, Lutz AK, Rousseaux MW, Bevilacqua L, Jahani-Asl A, Callaghan S, MacLaurin JG, Winklhofer KF (2010). Loss of the Parkinson's disease-linked gene DJ-1 perturbs mitochondrial dynamics. Human molecular genetics.

[29] Silvestri L, Caputo V, Bellacchio E, Atorino L, Dallapiccola B, Valente EM, Casari G (2005). Mitochondrial import and enzymatic activity of PINK1 mutants associated to recessive parkinsonism. Human molecular genetics.

[30] Zhou C, Huang Y, Shao Y, May J, Prou D, Perier C, Dauer W, Schon EA, Przedborski S (2008). The kinase domain of mitochondrial PINK1 faces the cytoplasm. Proceedings of the National Academy of Sciences of the United States of America.

[31] Warner TT, Schapira AH (2003). Genetic and environmental factors in the cause of Parkinson's disease. Annals of neurology.

[32] Geldenhuys WJ, Leeper TC, Carroll RT (2014). mitoNEET as a novel drug target for mitochondrial dysfunction. Drug discovery today.

[33] Beal MF (2003). Mitochondria, oxidative damage, and inflammation in Parkinson's disease. Annals of the New York Academy of Sciences.

[34] Ryu EJ, Harding HP, Angelastro JM, Vitolo OV, Ron D, Greene LA (2002). Endoplasmic reticulum stress and the unfolded protein response in cellular models of Parkinson's disease. The Journal of neuroscience: the official journal of the Society for Neuroscience.

[35] Pan PY, Yue Z (2014). Genetic causes of Parkinson's disease and their links to autophagy regulation. Parkinsonism & related disorders.

[36] Antony PM, Diederich NJ, Balling R (2011). Parkinson's disease mouse models in translational research. Mammalian genome: official journal of the International Mammalian Genome Society.

[37] Antony PM, Diederich NJ, Krüger R, Balling R (2013). The hallmarks of Parkinson's disease. The FEBS journal.

[38] Fujita KA, Ostaszewski M, Matsuoka Y, Ghosh S, Glaab E, Trefois C, Crespo I, Perumal TM, Jurkowski W, Antony PM, Diederich N, Buttini M, Kodama A (2014). Integrating pathways of Parkinson's disease in a molecular interaction map. Molecular neurobiology.

[39] Filiou MD, Arefin AS, Moscato P, Graeber MB (2014). 'Neuroinflammation' differs categorically from inflammation: transcriptomes of Alzheimer's disease, Parkinson's disease, schizophrenia and inflammatory diseases compared. Neurogenetics.

[40] Amor S, Peferoen LA, Vogel DY, Breur M, van der Valk P, Baker D, van Noort JM (2013). Inflammation in neurodegenerative diseases - an update. Immunology.

[41] Farooqui T, Farooqui AA (2011). Lipid-mediated oxidative stress and inflammation in the pathogenesis of Parkinson's disease. Parkinson's disease.

[42] Koziorowski D, Tomasiuk R, Szlufik S, Friedman A (2012). Inflammatory cytokines and NT-proCNP in Parkinson's disease patients. Cytokine.

[43] Kell DB (2009). Iron behaving badly: inappropriate iron chelation as a major contributor to the aetiology of vascular and other progressive inflammatory and degenerative diseases. BMC medical genomics.

[44] Kell DB (2010). Towards a unifying, systems biology understanding of large-scale cellular death and destruction caused by poorly liganded iron: Parkinson's, Huntington's, Alzheimer's, prions, bactericides, chemical toxicology and others as examples. Archives of toxicology.

[45] Kell DB, Pretorius E (2014). Serum ferritin is an important inflammatory disease marker, as it is mainly a leakage product from damaged cells. Metallomics.

[46] Kannarkat GT, Boss JM, Tansey MG (2013). The role of innate and adaptive immunity in Parkinson's disease. Journal of Parkinson's disease.

[47] Pessoa Rocha N, Reis HJ, Vanden Berghe P, Cirillo C (2014). Depression and cognitive impairment in Parkinson's disease: a role for inflammation and immunomodulation?. Neuroimmunomodulation.

[48] Deleidi M, Gasser T (2013). The role of inflammation in sporadic and familial Parkinson's disease. Cellular and molecular life sciences: CMLS.

[49] More SV, Kumar H, Kim IS, Song SY, Choi DK (2013). Cellular and molecular mediators of neuroinflammation in the pathogenesis of Parkinson's disease. Mediators of inflammation.

[50] Nolan YM, Sullivan AM, Toulouse A (2013). Parkinson's disease in the nuclear age of neuroinflammation. Trends in molecular medicine.

[51] Taylor JM, Main BS, Crack PJ (2013). Neuroinflammation and oxidative stress: co-conspirators in the pathology of Parkinson's disease. Neurochemistry international.

[52] Martinez-Martin P (2013). Instruments for holistic assessment of Parkinson's disease. Journal of neural transmission.

[53] Przuntek H, Muller T, Riederer P (2004). Diagnostic staging of Parkinson's disease: conceptual aspects. Journal of neural transmission.

[54] Lindqvist D, Hall S, Surova Y, Nielsen HM, Janelidze S, Brundin L, Hansson O (2013). Cerebrospinal fluid inflammatory markers in Parkinson's disease--associations with depression, fatigue, and cognitive impairment. Brain, behavior, and immunity.

[55] Song IU, Kim YD, Cho HJ, Chung SW (2013). Is neuroinflammation involved in the development of dementia in patients with Parkinson's disease?. Internal medicine (Tokyo, Japan).

[56] Kortekaas R, Leenders KL, van Oostrom JC, Vaalburg W, Bart J, Willemsen AT, Hendrikse NH (2005). Blood-brain barrier dysfunction in parkinsonian midbrain in vivo. Annals of neurology.

[57] Pisani V, Stefani A, Pierantozzi M, Natoli S, Stanzione P, Franciotta D, Pisani A (2012). Increased blood-cerebrospinal fluid transfer of albumin in advanced Parkinson's disease. Journal of neuroinflammation.

[58] Lee H, Pienaar IS (2014). Disruption of the blood-brain barrier in Parkinson's disease: curse or route to a cure?. Frontiers in bioscience (Landmark edition).

[59] Farkas E, De Jong GI, de Vos RA, Jansen Steur EN, Luiten PG (2000). Pathological features of cerebral cortical capillaries are doubled in Alzheimer's disease and Parkinson's disease. Acta neuropathologica.

[60] Wang J, Du XX, Jiang H, Xie JX (2009). Curcumin attenuates 6-hydroxydopamine-induced cytotoxicity by anti-oxidation and nuclear factor-kappa B modulation in MES23.5 cells. Biochemical pharmacology.

[61] Reelfs O, Tyrrell RM, Pourz C (2004). Ultraviolet a radiation-induced immediate iron release is a key modulator of the activation of NF-kappaB in human skin fibroblasts. The Journal of investigative dermatology.

[62] Asehnoune K, Strassheim D, Mitra S, Kim JY, Abraham E (2004). Involvement of reactive oxygen species in Toll-like receptor 4-dependent activation of NF-kappa, B. Journal of immunology.

[63] Gloire G, Legrand-Poels S, Piette J (2006). NF-kappaB activation by reactive oxygen species: fifteen years later. Biochemical pharmacology.

[64] Manna SK, Sarkar S, Barr J, Wise K, Barrera EV, Jejelowo O, Rice-Ficht AC, Ramesh GT (2005). Single-walled carbon nanotube induces oxidative stress and activates nuclear transcription factor-kappaB in human keratinocytes. Nano letters.

[65] Ashall L, Horton CA, Nelson DE, Paszek P, Harper CV, Sillitoe K, Ryan S, Spiller DG, Unitt JF, Broomhead DS, Kell DB, Rand DA, See V (2009). Pulsatile stimulation determines timing and specificity of NF-kappaB-dependent transcription. Science.

[66] Nelson DE, Ihekwaba AE, Elliott M, Johnson JR, Gibney CA, Foreman BE, Nelson G, See V, Horton CA, Spiller DG, Edwards SW, McDowell HP, Unitt JF (2004). Oscillations in NF-kappaB signaling control the dynamics of gene expression. Science.

[67] Sagai M, Bocci V (2011). Mechanisms of Action Involved in Ozone Therapy: Is healing induced via a mild oxidative stress?. Medical gas research.

[68] Shi J, Johansson J, Woodling NS, Wang Q, Montine TJ, Andreasson K (2010). The prostaglandin E2 E-prostanoid 4 receptor exerts anti-inflammatory effects in brain innate immunity. Journal of immunology.

[69] Liang X, Wu L, Wang Q, Hand T, Bilak M, McCullough L, Andreasson K (2007). Function of COX-2 and prostaglandins in neurological disease. Journal of molecular neuroscience: MN.

[70] Hernanz R, Briones AM, Salaices M, Alonso MJ (2014). New roles for old pathways?. A circuitous relationship between reactive oxygen species and cyclo-oxygenase in hypertension. Clinical science.

[71] Berk M, Dean O, Drexhage H, McNeil JJ, Moylan S, Oneil A, Davey CG, Sanna L, Maes M (2013). Aspirin: a review of its neurobiological properties and therapeutic potential for mental illness. BMC medicine.

[72] Ganesh T (2013). Prostanoid Receptor EP2 as a Therapeutic Target. Journal of medicinal chemistry.

[73] Teismann P (2012). COX-2 in the neurodegenerative process of Parkinson's disease. BioFactors (Oxford, England).

[74] Arnold S (2012). Cytochrome c oxidase and its role in neurodegeneration and neuroprotection. Advances in experimental medicine and biology.

[75] Phani S, Loike JD, Przedborski S (2012). Neurodegeneration and inflammation in Parkinson's disease. Parkinsonism & related disorders.

[76] Breyer RM, Bagdassarian CK, Myers SA, Breyer MD (2001). Prostanoid receptors: subtypes and signaling. Annual review of pharmacology and toxicology.

[77] Hata AN, Breyer RM (2004). Pharmacology and signaling of prostaglandin receptors: multiple roles in inflammation and immune modulation. Pharmacology & therapeutics.

[78] Tietz O, Marshall A, Wuest M, Wang M, Wuest F (2013). Radiotracers for molecular imaging of cyclooxygenase-2 (COX-2) enzyme. Curr Med Chem.

[79] Reffelmann T, Ittermann T, Dorr M, Volzke H, Reinthaler M, Petersmann A, Felix SB (2011). Low serum magnesium concentrations predict cardiovascular and all-cause mortality. Atherosclerosis.

[80] Cimino PJ, Keene CD, Breyer RM, Montine KS, Montine TJ (2008). Therapeutic targets in prostaglandin E2 signaling for neurologic disease. Curr Med Chem.

[81] Zhao H, Cheng L, Liu Y, Zhang W, Maharjan S, Cui Z, Wang X, Tang D, Nie L (2014). Mechanisms of anti-inflammatory property of conserved dopamine neurotrophic factor: inhibition of JNK signaling in lipopolysaccharide-induced microglia. Journal of molecular neuroscience: MN.

[82] McGeer PL, McGeer EG (2002). Innate immunity, local inflammation, and degenerative disease. Science of aging knowledge environment: SAGE KE.

[83] Sudlow AW, Carey F, Forder R, Rothwell NJ (1996). The role of lipocortin-1 in dexamethasone-induced suppression of PGE2 and TNF alpha release from human peripheral blood mononuclear cells. British journal of pharmacology.

[84] Norden DM, Fenn AM, Dugan A, Godbout JP (2014). TGFbeta produced by IL-10 redirected astrocytes attenuates microglial activation. Glia.

[85] Meraz-Rios MA, Toral-Rios D, Franco-Bocanegra D, Villeda-Hernandez J, Campos-Pena V (2013). Inflammatory process in Alzheimer's Disease. Frontiers in integrative neuroscience.

[86] Saijo K, Winner B, Carson CT, Collier JG, Boyer L, Rosenfeld MG, Gage FH, Glass CK (2009). A Nurr1/CoREST pathway in microglia and astrocytes protects dopaminergic neurons from inflammation-induced death. Cell.

[87] De Astis S, Corradini I, Morini R, Rodighiero S, Tomasoni R, Lenardi C, Verderio C, Milani P, Matteoli M (2013). Nanostructured TiO2 surfaces promote polarized activation of microglia, but not astrocytes, toward a proinflammatory profile. Nanoscale.

[88] Andreasson K (2010). Emerging roles of PGE2 receptors in models of neurological disease. Prostaglandins & other lipid mediators.

[89] Liang X, Wang Q, Hand T, Wu L, Breyer RM, Montine TJ, Andreasson K (2005). Deletion of the prostaglandin E2 EP2 receptor reduces oxidative damage and amyloid burden in a model of Alzheimer's disease. The Journal of neuroscience: the official journal of the Society for Neuroscience.

[90] Tzeng SF, Hsiao HY, Mak OT (2005). Prostaglandins and cyclooxygenases in glial cells during brain inflammation. Current drug targets Inflammation and allergy.

[91] Rivest S (2010). Interactions between the immune and neuroendocrine systems. Progress in brain research.

[92] Montine TJ, Milatovic D, Gupta RC, Valyi-Nagy T, Morrow JD, Breyer RM (2002). Neuronal oxidative damage from activated innate immunity is EP2 receptor-dependent. Journal of neurochemistry.

[93] Liang X, Wang Q, Shi J, Lokteva L, Breyer RM, Montine TJ, Andreasson K (2008). The prostaglandin E2 EP2 receptor accelerates disease progression and inflammation in a model of amyotrophic lateral sclerosis. Annals of neurology.

[94] Lin H, Li HF, Lian WS, Chen HH, Lan YF, Lai PF, Cheng CF (2013). Thromboxane A2 mediates iron-overload cardiomyopathy in mice through calcineurin-nuclear factor of activated T cells signaling pathway. Circulation journal: official journal of the Japanese Circulation Society.

[95] Li X, Cudaback E, Breyer RM, Montine KS, Keene CD, Montine TJ (2012). Eicosanoid receptor subtype-mediated opposing regulation of TLR-stimulated expression of astrocyte glial-derived neurotrophic factor. FASEB journal: official publication of the Federation of American Societies for Experimental Biology.

[96] Kubo K, Inada T, Shingu K (2011). Possible role of propofol's cyclooxygenase-inhibiting property in alleviating dopaminergic neuronal loss in the substantia nigra in an MPTP-induced murine model of Parkinson's disease. Brain research.

[97] Dolegowska B, Lubkowska A, De Girolamo L (2012). Platelet lipidomic. Journal of biological regulators and homeostatic agents.

[98] Wypijewska A, Galazka-Friedman J, Bauminger ER, Wszolek ZK, Schweitzer KJ, Dickson DW, Jaklewicz A, Elbaum D, Friedman A (2010). Iron and reactive oxygen species activity in parkinsonian substantia nigra. Parkinsonism & related disorders.

[99] Jin L, Wang J, Jin H, Fei G, Zhang Y, Chen W, Zhao L, Zhao N, Sun X, Zeng M, Zhong C (2012). Nigral iron deposition occurs across motor phenotypes of Parkinson's disease. European journal of neurology: the official journal of the European Federation of Neurological Societies.

[100] Wang C, Fan G, Xu K, Wang S (2013). Quantitative assessment of iron deposition in the midbrain using 3D-enhanced T2 star weighted angiography (ESWAN): a preliminary cross-sectional study of 20 Parkinson's disease patients. Magnetic resonance imaging.

[101] Double KL (2012). Neuronal vulnerability in Parkinson's disease. Parkinsonism & related disorders.

[102] Double KL, Halliday GM, Dunkley PR, Dickson PW, Gerlach M, Riederer P (2010). Pigmentation in the human brain and risk of Parkinson's disease. Annals of neurology.

[103] Ouazia D, Levros LC, Rassart E, Desrosiers RR (2014). Dopamine down-regulation of protein L-isoaspartyl methyltransferase is dependent on reactive oxygen species in SH-SY5Y cells. Neuroscience.

[104] Lopert P, Patel M (2014). Nicotinamide nucleotide transhydrogenase (Nnt) links the substrate requirement in brain mitochondria for hydrogen peroxide removal to the thioredoxin/peroxiredoxin (Trx/Prx) system. The Journal of biological chemistry.

[105] Milatovic D, Gupta RC, Yu Y, Zaja-Milatovic S, Aschner M (2011). Protective effects of antioxidants and anti-inflammatory agents against manganese-induced oxidative damage and neuronal injury. Toxicology and applied pharmacology.

[106] Mosley RL, Benner EJ, Kadiu I, Thomas M, Boska MD, Hasan K, Laurie C, Gendelman HE (2006). Neuroinflammation, Oxidative Stress and the Pathogenesis of Parkinson's Disease. Clinical neuroscience research.

[107] Altamura S, Muckenthaler MU (2009). Iron toxicity in diseases of aging: Alzheimer's disease, Parkinson's disease and atherosclerosis. Journal of Alzheimer's disease: JAD.

[108] Barnham KJ, Bush AI (2008). Metals in Alzheimer's and Parkinson's diseases. Current opinion in chemical biology.

[109] Brewer GJ (2007). Iron and copper toxicity in diseases of aging, particularly atherosclerosis and Alzheimer's disease. Experimental biology and medicine (Maywood, NJ).

[110] Crichton RR, Dexter DT, Ward RJ (2011). Brain iron metabolism and its perturbation in neurological diseases. Journal of neural transmission.

[111] Friedman A, Arosio P, Finazzi D, Koziorowski D, Galazka-Friedman J (2011). Ferritin as an important player in neurodegeneration. Parkinsonism & related disorders.

[112] Gotz ME, Double K, Gerlach M, Youdim MB, Riederer P (2004). The relevance of iron in the pathogenesis of Parkinson's disease. Annals of the New York Academy of Sciences.

[113] Jomova K, Vondrakova D, Lawson M, Valko M (2010). Metals, oxidative stress and neurodegenerative disorders. Molecular and cellular biochemistry.

[114] Ke Y, Ming Qian Z (2003). Iron misregulation in the brain: a primary cause of neurodegenerative disorders. Lancet neurology.

[115] Núñez MT, Urrutia P, Mena N, Aguirre P, Tapia V, Salazar J (2012). Iron toxicity in neurodegeneration. Biometals: an international journal on the role of metal ions in biology, biochemistry, and medicine.

[116] Schneider SA, Bhatia KP (2012). Syndromes of neurodegeneration with brain iron accumulation. Seminars in pediatric neurology.

[117] Schneider SA, Hardy J, Bhatia KP (2012). Syndromes of neurodegeneration with brain iron accumulation (NBIA): an update on clinical presentations, histological and genetic underpinnings, and treatment considerations. Movement disorders: official journal of the Movement Disorder Society.

[118] Stankiewicz JM, Brass SD (2009). Role of iron in neurotoxicity: a cause for concern in the elderly?. Current opinion in clinical nutrition and metabolic care.

[119] Thomas M, Jankovic J (2004). Neurodegenerative disease and iron storage in the brain. Current opinion in neurology.

[120] Berg D (2007). Disturbance of iron metabolism as a contributing factor to SN hyperechogenicity in Parkinson's disease: implications for idiopathic and monogenetic forms. Neurochemical research.

[121] Aamodt AH, Stovner LJ, Thorstensen K, Lydersen S, White LR, Aasly JO (2007). Prevalence of haemochromatosis gene mutations in Parkinson's disease. Journal of neurology, neurosurgery, and psychiatry.

[122] Bartzokis G, Tishler TA, Lu PH, Villablanca P, Altshuler LL, Carter M, Huang D, Edwards N, Mintz J (2007). Brain ferritin iron may influence age- and gender-related risks of neurodegeneration. Neurobiology of aging.

[123] Farhoudi M, Taheraghdam A, Farid GA, Talebi M, Pashapou A, Majidi J, Goldust M (2012). Serum iron and ferritin level in idiopathic Parkinson. Pakistan journal of biological sciences: PJBS.

[124] Singh N, Haldar S, Tripathi AK, McElwee MK, Horback K, Beserra A (2014). Iron in Neurodegenerative Disorders of Protein Misfolding: A Case of Prion Disorders and Parkinson's Disease. Antioxidants & redox signaling.

[125] Schneider SA, Bhatia KP (2013). Excess iron harms the brain: the syndromes of neurodegeneration with brain iron accumulation (NBIA). Journal of neural transmission.

[126] Hare D, Ayton S, Bush A, Lei P (2013). A delicate balance: Iron metabolism and diseases of the brain. Frontiers in aging neuroscience.

[127] Boelmans K, Holst B, Hackius M, Finsterbusch J, Gerloff C, Fiehler J, Münchau A (2012). Brain iron deposition fingerprints in Parkinson's disease and progressive supranuclear palsy. Movement disorders: official journal of the Movement Disorder Society.

[128] Han YH, Lee JH, Kang BM, Mun CW, Baik SK, Shin YI, Park KH (2013). Topographical differences of brain iron deposition between progressive supranuclear palsy and parkinsonian variant multiple system atrophy. Journal of the neurological sciences.

[129] Double KL, Gerlach M, Youdim MB, Riederer P (2000). Impaired iron homeostasis in Parkinson's disease. Journal of neural transmission Supplementum.

[130] Faucheux BA, Martin ME, Beaumont C, Hauw JJ, Agid Y, Hirsch EC (2003). Neuromelanin associated redox-active iron is increased in the substantia nigra of patients with Parkinson's disease. Journal of neurochemistry.

[131] Lewis MM, Du G, Kidacki M, Patel N, Shaffer ML, Mailman RB, Huang X (2013). Higher iron in the red nucleus marks Parkinson's dyskinesia. Neurobiology of aging.

[132] Mariani S, Ventriglia M, Simonelli I, Spalletta G, Bucossi S, Siotto M, Assogna F, Melgari JM, Vernieri F, Squitti R (2013). Effects of hemochromatosis and transferrin gene mutations on peripheral iron dyshomeostasis in mild cognitive impairment and Alzheimer's and Parkinson's diseases. Frontiers in aging neuroscience.

[133] Rossi M, Ruottinen H, Soimakallio S, Elovaara I, Dastidar P (2013). Clinical MRI for iron detection in Parkinson's disease. Clinical imaging.

[134] Berg D, Hochstrasser H (2006). Iron metabolism in Parkinsonian syndromes. Movement disorders: official journal of the Movement Disorder Society.

[135] Berg D, Youdim MB (2006). Role of iron in neurodegenerative disorders. Topics in magnetic resonance imaging: TMRI.

[136] Kasinathan RS, Föller M, Koka S, Huber SM, Lang F (2007). Inhibition of eryptosis and intraerythrocytic growth of Plasmodium falciparum by flufenamic acid. Naunyn-Schmiedeberg's archives of pharmacology.

[137] Skretteberg PT, Bodegård J, Kjeldsen SE, Erikssen G, Thaulow E, Sandvik L, Erikssen JE (2010). Interaction between inflammation and blood viscosity predicts cardiovascular mortality. Scandinavian cardiovascular journal: SCJ.

[138] Arreguin S, Nelson P, Padway S, Shirazi M, Pierpont C (2009). Dopamine complexes of iron in the etiology and pathogenesis of Parkinson's disease. Journal of inorganic biochemistry.

[139] Devos D, Moreau C, Devedjian JC, Kluza J, Petrault M, Laloux C, Jonneaux A, Ryckewaert G, Garcon G, Rouaix N, Duhamel A, Jissendi P, Dujardin K (2014). Targeting Chelatable Iron as a Therapeutic Modality in Parkinson's Disease. Antioxidants & redox signaling.

[140] Gałązka-Friedman J, Friedman A (2011). Role of iron in pathogenesis of Parkinson disease. Biotechnologia.

[141] Berg D, Riederer P, Gerlach M (2008). Contribution of disturbed iron metabolism to the pathogenesis of Parkinson's disease. Future Medicine.

[142] Dukovski D, Li Z, Kelly DF, Mack E, Walz T (2009). Structural and functional studies on the stalk of the transferrin receptor. Biochemical and biophysical research communications.

[143] Madenci G, Bilen S, Arli B, Saka M, Ak F (2012). Serum iron, vitamin B12 and folic acid levels in Parkinson's disease. Neurochemical research.

[144] Tórsdóttir G, Kristinsson J, Sveinbjörnsdóttir S, Snaedal J, Jóhannesson T (1999). Copper, ceruloplasmin, superoxide dismutase and iron parameters in Parkinson's disease. Pharmacology & toxicology.

[145] Logroscino G, Marder K, Graziano J, Freyer G, Slavkovich V, LoIacono N, Cote L, Mayeux R (1997). Altered systemic iron metabolism in Parkinson's disease. Neurology.

[146] Wu WH, Meydani M, Meydani SN, Burklund PM, Blumberg JB, Munro HN (1990). Effect of dietary iron overload on lipid peroxidation, prostaglandin synthesis and lymphocyte proliferation in young and old rats. The Journal of nutrition.

[147] Zwart SR, Morgan JL, Smith SM (2013). Iron status and its relations with oxidative damage and bone loss during long-duration space flight on the International Space Station. The American journal of clinical nutrition.

[148] Yanai TK, Mori S (2008). Density functional studies on thromboxane biosynthesis: mechanism and role of the heme-thiolate system. Chemistry, an Asian journal.

[149] Cheng CF, Lian WS (2013). Prooxidant mechanisms in iron overload cardiomyopathy. BioMed research international.

[150] Undas A (2014). Fibrin clot properties and their modulation in thrombotic disorders. Thrombosis and haemostasis.

[151] Undas A (2011). Acquired dysfibrinogenemia in atherosclerotic vascular disease. Polskie Archiwum Medycyny Wewnetrznej.

[152] Undas A, Ariëns RA (2011). Fibrin clot structure and function: a role in the pathophysiology of arterial and venous thromboembolic diseases. Arteriosclerosis, thrombosis, and vascular biology.

[153] Pretorius E, Kell DB (2014). Diagnostic morphology: biophysical indicators for iron-driven inflammatory diseases. Integrative Biology.

[154] Pretorius E, Bester J, Vermeulen N, Lipinski B, Gericke GS, Kell DB (2014). Profound morphological changes in the erythrocytes and fibrin networks of patients with hemochromatosis or with hyperferritinemia, and their normalization by iron chelators and other agents. PlosOne.

[155] Pretorius E, Lipinski B (2012). Differences in Morphology of Fibrin Clots Induced with Thrombin and Ferric Ions and Its Pathophysiological Consequences. Heart, lung & circulation.

[156] Pretorius E, Oberholzer HM, van der Spuy WJ, Meiring JH (2010). Smoking and coagulation: the sticky fibrin phenomenon. Ultrastruct Pathol.

[157] Pretorius E, Oberholzer HM, van der Spuy WJ, Swanepoel AC, Soma P (2011). Qualitative scanning electron microscopy analysis of fibrin networks and platelet abnormalities in diabetes. Blood coagulation & fibrinolysis: an international journal in haemostasis and thrombosis.

[158] Sostres C, Gargallo CJ, Lanas A (2014). Aspirin, cyclooxygenase inhibition and colorectal cancer. World journal of gastrointestinal pharmacology and therapeutics.

[159] Santilli F, Vazzana N, Liani R, Guagnano MT, Davi G (2012). Platelet activation in obesity and metabolic syndrome. Obesity reviews: an official journal of the International Association for the Study of Obesity.

[160] Vanhoutte PM (2009). COX-1 and vascular disease. Clinical pharmacology and therapeutics.

[161] Casoli T, Di Stefano G, Balietti M, Solazzi M, Giorgetti B, Fattoretti P (2010). Peripheral inflammatory biomarkers of Alzheimer's disease: the role of platelets. Biogerontology.

[162] Goltsov A, Maryashkin A, Swat M, Kosinsky Y, Humphery-Smith I, Demin O, Goryanin I, Lebedeva G (2009). Kinetic modelling of NSAID action on COX-1: focus on in vitro/in vivo aspects and drug combinations. European journal of pharmaceutical sciences: official journal of the European Federation for Pharmaceutical Sciences.

[163] Valles J, Lago A, Moscardo A, J IT, Parkhutik V, Santos MT (2013). TXA2 synthesis and COX1-independent platelet reactivity in aspirin-treated patients soon after acute cerebral stroke or transient ischaemic attack. Thrombosis research.

[164] Goggs R, Poole AW (2012). Platelet signaling-a primer. Journal of veterinary emergency and critical care.

[165] Knebel SM, Elrick MM, Bowles EA, Zdanovec AK, Stephenson AH, Ellsworth ML, Sprague RS (2013). Synergistic effects of prostacyclin analogs and phosphodiesterase inhibitors on cyclic adenosine 3′,5′ monophosphate accumulation and adenosine 3′5′ triphosphate release from human erythrocytes. Experimental biology and medicine (Maywood, NJ).

[166] Angiolillo DJ, Ferreiro JL (2013). Antiplatelet and anticoagulant therapy for atherothrombotic disease: the role of current and emerging agents. American journal of cardiovascular drugs: drugs, devices, and other interventions.

[167] Rao AK (2013). Inherited platelet function disorders: overview and disorders of granules, secretion, and signal transduction. Hematology/oncology clinics of North America.

[168] Manolis AS, Manolis TA, Papadimitriou P, Koulouris S, Melita H (2013). Combined antiplatelet therapy: still a sweeping combination in cardiology. Cardiovascular & hematological agents in medicinal chemistry.

[169] de Souza Brito F, Tricoci P (2013). Novel anti-platelet agents: focus on thrombin receptor antagonists. Journal of cardiovascular translational research.

[170] Nurden A, Nurden P (2011). Advances in our understanding of the molecular basis of disorders of platelet function. Journal of thrombosis and haemostasis: JTH.

[171] Li Z, Delaney MK, O'Brien KA, Du X (2010). Signaling during platelet adhesion and activation. Arteriosclerosis, thrombosis, and vascular biology.

[172] Santos MT, Valles J, Lago A, Tembl J, Sanchez E, Moscardo A, Cosin J (2008). Residual platelet thromboxane A2 and prothrombotic effects of erythrocytes are important determinants of aspirin resistance in patients with vascular disease. Journal of thrombosis and haemostasis: JTH.

[173] Santos MT, Valles J, Marcus AJ, Safier LB, Broekman MJ, Islam N, Ullman HL, Eiroa AM, Aznar J (1991). Enhancement of platelet reactivity and modulation of eicosanoid production by intact erythrocytes. A new approach to platelet activation and recruitment. The Journal of clinical investigation.

[174] Gudjoncik A, Guenancia C, Zeller M, Cottin Y, Vergely C, Rochette L (2014). Iron, oxidative stress, and redox signaling in the cardiovascular system. Molecular nutrition & food research.

[175] Ahmed MS, Jadhav AB, Hassan A, Meng QH (2012). Acute Phase Reactants as Novel Predictors of Cardiovascular Disease. ISRN inflammation.

[176] Bester J, Buys AV, Lipinski B, Kell DB, Pretorius E (2013). High ferritin levels have major effects on the morphology of erythrocytes in Alzheimer's disease. Frontiers in aging neuroscience.

[177] Buys AV, Van Rooy MJ, Soma P, Van Papendorp D, Lipinski B, Pretorius E (2013). Changes in red blood cell membrane structure in type 2 diabetes: a scanning electron and atomic force microscopy study. Cardiovascular diabetology.

[178] Lipinski B, Pretorius E (2012). Novel pathway of ironinduced blood coagulation: implications for diabetes mellitus and its complications. Polskie Archiwum Medycyny Wewnetrznej.

[179] Pretorius E, Lipinski B, Bester J, Vermeulen N, Soma P (2013). Albumin stabilizes fibrin fiber ultrastructure in low serum albumin type 2 diabetes. Ultrastruct Pathol.

[180] Pretorius E, Briedenhann S, Marx J, Franz RC (2006). Structural changes in the fibrin network of a pretoria family with dysfibrinogenemia: a scanning electron microscopical study. Ultrastruct Pathol.

[181] Pretorius E, Oberholzer HM, Smit E, Steyn E, Briedenhann S, Franz CR (2008). Ultrastructural changes in platelet aggregates of HIV patients: a scanning electron microscopy study. Ultrastruct Pathol.

[182] Pretorius E, Smit E, Oberholzer HM, Steyn E, Briedenhann S, Franz RC (2009). Investigating the ultrastructure of platelets of HIV patients treated with the immuno-regulator, Canova: a qualitative scanning electron microscopy study. Histology and histopathology.

[183] Pretorius E, Vermeulen N, Bester J, du Plooy JL, Gericke GS (2014). Extreme iron overload and the effect on red blood cell morphology. The Lancet.

[184] Pretorius E (2012). Ultrastructural changes in platelet membranes due to cigarette smoking. Ultrastruct Pathol.

[185] Pretorius E, du Plooy JN, Soma P, Keyser I, Buys AV (2013). Smoking and fluidity of erythrocyte membranes: A high resolution scanning electron and atomic force microscopy investigation. Nitric oxide: biology and chemistry / official journal of the Nitric Oxide Society.

[186] Gasparyan AY, Ayvazyan L, Pretorius E, Kitas GD (2013). Platelets in Rheumatic Diseases: Friend or Foe?. Current pharmaceutical design.

[187] Pretorius E, du Plooy J, Soma P, Gasparyan AY (2013). An ultrastructural analysis of platelets, erythrocytes, white blood cells, and fibrin network in systemic lupus erythematosus. Rheumatology international.

[188] Pretorius E, Oberholzer HM, van der Spuy WJ, Swanepoel AC, Soma P (2012). Scanning electron microscopy of fibrin networks in rheumatoid arthritis: a qualitative analysis. Rheumatology international.

[189] Pretorius E, Ekpo OE, Smit E (2007). Comparative ultrastructural analyses of platelets and fibrin networks using the murine model of asthma. Experimental and toxicologic pathology: official journal of the Gesellschaft fur Toxikologische Pathologie.

[190] Pretorius E, Oberholzer HM, Vieira WA, Smit E (2009). Ultrastructure of platelets and fibrin networks of asthmatic mice exposed to selenium and Withania somnifera. Anatomical science international.

[191] Pretorius EV, N.; Bester J (2013). Atypical erythrocytes and platelets in a patient with a pro-thrombin mutation. Platelets.

[192] Pretorius E, Humphries P (2007). Ultrastructural changes to rabbit fibrin and platelets due to aspartame. Ultrastruct Pathol.

[193] van der Spuy WJ, Pretorius E (2013). Interaction of red blood cells adjacent to and within a thrombus in experimental cerebral ischaemia. Thrombosis research.

[194] Van Der Spuy WJ, Pretorius E (2013). A place for ultrastructural analysis of platelets in cerebral ischemic research. Microsc Res Tech.

[195] Lipinski B, Pretorius E (2012). Hydroxyl radical-modified fibrinogen as a marker of thrombosis: the role of iron. Hematology.

[196] Lipinski B, Pretorius E (2013). Iron-Induced Fibrin in Cardiovascular Disease. Current neurovascular research.

[197] Lipinski B, Pretorius E, Oberholzer HM, Van Der Spuy WJ (2012). Iron enhances generation of fibrin fibers in human blood: Implications for pathogenesis of stroke. Microsc Res Tech.

[198] Pretorius E (2011). Quantifying changes in fibrin fiber network morphology. Ultrastruct Pathol.

[199] Pretorius E, Lipinski B (2013). Thromboembolic ischemic stroke changes red blood cell morphology. Cardiovascular pathology: the official journal of the Society for Cardiovascular Pathology.

[200] Pretorius E, Steyn H, Engelbrecht M, Swanepoel AC, Oberholzer HM (2011). Differences in fibrin fiber diameters in healthy individuals and thromboembolic ischemic stroke patients. Blood coagulation & fibrinolysis: an international journal in haemostasis and thrombosis.

[201] Swanepoel AC, Pretorius E (2012). Scanning electron microscopy analysis of erythrocytes in thromboembolic ischemic stroke. International journal of laboratory hematology.

[202] Pretorius E, Lipinski B (2013). Iron alters red blood cell morphology. Blood.

[203] Pretorius E, Vermeulen N, Bester J, Lipinski B (2013). Novel use of scanning electron microscopy for detection of iron-induced morphological changes in human blood. Microsc Res Tech.

[204] Pretorius E, Bester J, Vermeulen N, Lipinski B (2013). Oxidation inhibits iron-induced blood coagulation. Current drug targets.

[205] Azizova OA, Shvachko AG, Aseichev AV (2009). Effect of iron ions on functional activity of thrombin. Bulletin of experimental biology and medicine.

[206] Lipinski B, Pretorius E, Oberholzer HM, van der Spuy WJ (2012). Interaction of fibrin with red blood cells: the role of iron. Ultrastruct Pathol.

[207] Olinescu RM, Kummerow FA (2001). Fibrinogen is an efficient antioxidant. The Journal of nutritional biochemistry.

[208] Feng YH, Hart G (1995). In vitro oxidative damage to tissue-type plasminogen activator: a selective modification of the biological functions. Cardiovascular research.

[209] Upchurch GR, Ramdev N, Walsh MT, Loscalzo J (1998). Prothrombotic Consequences of the Oxidation of Fibrinogen and their Inhibition by Aspirin. Journal of thrombosis and thrombolysis.

[210] Undas A, Szuldrzynski K, Stepien E, Zalewski J, Godlewski J, Tracz W, Pasowicz M, Zmudka K (2008). Reduced clot permeability and susceptibility to lysis in patients with acute coronary syndrome: effects of inflammation and oxidative stress. Atherosclerosis.

[211] Schwartz RS, Halliday GM, Cordato DJ, Kril JJ (2012). Small-vessel disease in patients with Parkinson's disease: a clinicopathological study. Movement disorders: official journal of the Movement Disorder Society.

[212] Mastaglia FL, Johnsen RD, Kakulas BA (2002). Prevalence of stroke in Parkinson's disease: a postmortem study. Movement disorders: official journal of the Movement Disorder Society.

[213] Garcia-Gracia C, Khan T, Reyes D, McMahan S, Galvez-Jimenez N (2013). Conference presentation: The Prevalence of Stroke in Parkinson's Disease Is High: A Risk Factor Assessment. Neurology.

[214] Huang YP, Chen LS, Yen MF, Fann CY, Chiu YH, Chen HH, Pan SL (2013). Parkinson's disease is related to an increased risk of ischemic stroke-a population-based propensity score-matched follow-up study. PLoS One.

[215] Föller M, Huber SM, Lang F (2008). Erythrocyte programmed cell death. IUBMB life.

[216] Lang E, Qadri SM, Lang F (2012). Killing me softly - suicidal erythrocyte death. The international journal of biochemistry & cell biology.

[217] Lang F, Abed M, Lang E, Föller M (2013). Oxidative stress and suicidal erythrocyte death. Antioxidants & redox signaling.

[218] Lang F, Lang E, Föller M (2012). Physiology and pathophysiology of eryptosis. Transfusion medicine and hemotherapy: offizielles Organ der Deutschen Gesellschaft fur Transfusionsmedizin und Immunhamatologie.

[219] Lang F, Qadri SM (2012). Mechanisms and significance of eryptosis, the suicidal death of erythrocytes. Blood purification.

[220] Bissinger R, Modicano P, Frauenfeld L, Lang E, Jacobi J, Faggio C, Lang F (2013). Estramustine-induced suicidal erythrocyte death. Cellular physiology and biochemistry: international journal of experimental cellular physiology, biochemistry, and pharmacology.

[221] Lang NN, Connelly DT (2013). Novel oral anticoagulants for the prevention of thromboembolism in patients with atrial fibrillation. The journal of the Royal College of Physicians of Edinburgh.

[222] Gao M, Lau PM, Kong SK (2014). Mitochondrial toxin betulinic acid induces in vitro eryptosis in human red blood cells through membrane permeabilization. Archives of toxicology.

[223] Walker B, Towhid ST, Schmid E, Hoffmann SM, Abed M, Munzer P, Vogel S, Neis F, Brucker S, Gawaz M, Borst O, Lang F (2014). Dynamic adhesion of eryptotic erythrocytes to immobilized platelets via platelet phosphatidylserine receptors. American journal of physiology Cell physiology.

[224] Chakraborti S, Alam MN, Paik D, Shaikh S, Chakraborti T (2012). Implications of calpains in health and diseases. Indian journal of biochemistry & biophysics.

[225] Diepenbroek M, Casadei N, Esmer H, Saido TC, Takano J, Kahle PJ, Nixon RA, Rao MV, Melki R, Pieri L, Helling S, Marcus K, Krueger R (2014). Overexpression of the calpain-specific inhibitor calpastatin reduces human alpha-Synuclein processing, aggregation and synaptic impairment in alphaSyn transgenic mice. Human molecular genetics.

[226] Samantaray S, Knaryan VH, Shields DC, Banik NL (2013). Critical role of calpain in spinal cord degeneration in Parkinson's disease. Journal of neurochemistry.

[227] Kim C, Yun N, Lee YM, Jeong JY, Baek JY, Song HY, Ju C, Youdim MB, Jin BK, Kim WK, Oh YJ (2013). Gel-based protease proteomics for identifying the novel calpain substrates in dopaminergic neuronal cell. The Journal of biological chemistry.

[228] Arshad A, Chen X, Cong Z, Qing H, Deng Y (2014). TRPC1 protects dopaminergic SH-SY5Y cells from MPP+, salsolinol, and N-methyl-(R)-salsolinol-induced cytotoxicity. Acta Biochim Biophys Sin (Shanghai).

[229] Mattson MP (2007). Calcium and neurodegeneration. Aging cell.

[230] Arduino DM, Esteves AR, Cardoso SM, Oliveira CR (2009). Endoplasmic reticulum and mitochondria interplay mediates apoptotic cell death: relevance to Parkinson's disease. Neurochemistry international.

[231] Lang F, Gulbins E, Lang PA, Zappulla D, Föller M (2010). Ceramide in suicidal death of erythrocytes. Cellular physiology and biochemistry: international journal of experimental cellular physiology, biochemistry, and pharmacology.

[232] Kempe DS, Akel A, Lang PA, Hermle T, Biswas R, Muresanu J, Friedrich B, Dreischer P, Wolz C, Schumacher U, Peschel A, Götz F, Döring G (2007). Suicidal erythrocyte death in sepsis. Journal of molecular medicine (Berlin, Germany).

[233] Kolev OI, Pedersson S, Nilsson G, Tibbling L (1997). Cold caloric microcirculatory reflex disturbance in patients with Parkinson's disease. Clinical autonomic research: official journal of the Clinical Autonomic Research Society.

[234] Ben-Shachar D, Youdim MB (1991). Intranigral iron injection induces behavioral and biochemical “parkinsonism” in rats. Journal of neurochemistry.

[235] Kaur D, Yantiri F, Rajagopalan S, Kumar J, Mo JQ, Boonplueang R, Viswanath V, Jacobs R, Yang L, Beal MF, DiMonte D, Volitaskis I, Ellerby L (2003). Genetic or pharmacological iron chelation prevents MPTP-induced neurotoxicity in vivo: a novel therapy for Parkinson's disease. Neuron.

[236] Weinreb O, Mandel S, Youdim MB, Amit T (2013). Targeting dysregulation of brain iron homeostasis in Parkinson's disease by iron chelators. Free radical biology & medicine.

[237] Dexter DT, Statton SA, Whitmore C, Freinbichler W, Weinberger P, Tipton KF, Della Corte L, Ward RJ, Crichton RR (2011). Clinically available iron chelators induce neuroprotection in the 6-OHDA model of Parkinson's disease after peripheral administration. Journal of neural transmission.

[238] Gogoi S, Antonio T, Rajagopalan S, Reith M, Andersen J, Dutta AK (2011). Dopamine D(2)/D(3) agonists with potent iron chelation, antioxidant and neuroprotective properties: potential implication in symptomatic and neuroprotective treatment of Parkinson's disease. ChemMedChem.

[239] Perez CA, Tong Y, Guo M (2008). Iron Chelators as Potential Therapeutic Agents for Parkinson's Disease. Current bioactive compounds.

[240] Youdim MB, Stephenson G, Ben Shachar D (2004). Ironing iron out in Parkinson's disease and other neurodegenerative diseases with iron chelators: a lesson from 6-hydroxydopamine and iron chelators, desferal and VK-28. Annals of the New York Academy of Sciences.

[241] Youdim MB, Fridkin M, Zheng H (2004). Novel bifunctional drugs targeting monoamine oxidase inhibition and iron chelation as an approach to neuroprotection in Parkinson's disease and other neurodegenerative diseases. Journal of neural transmission.

[242] Mastroberardino PG, Hoffman EK, Horowitz MP, Betarbet R, Taylor G, Cheng D, Na HM, Gutekunst CA, Gearing M, Trojanowski JQ, Anderson M, Chu CT, Peng J (2009). A novel transferrin/TfR2-mediated mitochondrial iron transport system is disrupted in Parkinson's disease. Neurobiology of disease.

[243] Horowitz MP, Greenamyre JT (2010). Gene-environment interactions in Parkinson's disease: the importance of animal modeling. Clinical pharmacology and therapeutics.

[244] Boddaert N, Le Quan Sang KH, Rotig A, Leroy-Willig A, Gallet S, Brunelle F, Sidi D, Thalabard JC, Munnich A, Cabantchik ZI (2007). Selective iron chelation in Friedreich ataxia: biologic and clinical implications. Blood.

[245] Hoehn MM, Yahr MD (1967). Parkinsonism: onset, progression and mortality. Neurology.

[246] Stocchi F, Carbone A, Inghilleri M, Monge A, Ruggieri S, Berardelli A, Manfredi M (1997). Urodynamic and neurophysiological evaluation in Parkinson's disease and multiple system atrophy. Journal of neurology, neurosurgery, and psychiatry.

[247] Schrag A, Selai C, Jahanshahi M, Quinn NP (2000). The EQ-5D--a generic quality of life measure-is a useful instrument to measure quality of life in patients with Parkinson's disease. Journal of neurology, neurosurgery, and psychiatry.

[248] Schrag A, Jahanshahi M, Quinn N (2000). What contributes to quality of life in patients with Parkinson's disease?. Journal of neurology, neurosurgery, and psychiatry.

[249] Rodríguez-Violante M, Camacho-Ordoñez A, Cervantes-Arriaga A, González-Latapí P, Velázquez-Osuna S (2014). Factors associated with the quality of life of subjects with Parkinson's disease and burden on their caregivers. Neurologia (Barcelona, Spain).

[250] Karlsen KH, Tandberg E, Arsland D, Larsen JP (2000). Health related quality of life in Parkinson's disease: a prospective longitudinal study. Journal of neurology, neurosurgery, and psychiatry.

[251] Dufrêne YF, Martínez-Martin D, Medalsy I, Alsteens D, Müller DJ (2013). Multiparametric imaging of biological systems by force-distance curve-based AFM. Nature methods.

[252] Kolar P, Tomankova K, Malohlava J, Zapletalova J, Vujtek M, Safarova K, Jancik D, Kolarova H (2013). The effect of photodynamic treatment on the morphological and mechanical properties of the HeLa cell line. General physiology and biophysics.

[253] Berquand A (2011). Quantitative Imaging of Living Biological Samples by PeakForce QNM Atomic Force Microscopy. Bruker Application Note.

[254] Derjaguin B, Muller V, Toporov Y (1975). Effect of contact deformations on the adhesion of particles. J Colloid Interf Sci.

[255] Broadhurst D, Kell DB (2006). Statistical strategies for avoiding false discoveries in metabolomics and related experiments. Metabolomics.

[256] Sprenger FS, Seppi K, Poewe W (2012). Drug safety evaluation of rotigotine. Expert opinion on drug safety.

[257] Sanford M, Scott LJ (2011). Rotigotine transdermal patch: a review of its use in the treatment of Parkinson's disease. CNS drugs.

[258] Rascol O, Lozano A, Stern M, Poewe W (2011). Milestones in Parkinson's disease therapeutics. Movement disorders: official journal of the Movement Disorder Society.

[259] Stocchi F, Radicati FG, Torti M (2014). Drug safety evaluation of ropinirole prolonged release. Expert opinion on drug safety.

[260] Yun JY, Kim HJ, Lee JY, Kim YE, Kim JS, Kim JM, Jeon BS (2013). Comparison of once-daily versus twice-daily combination of ropinirole prolonged release in Parkinson's disease. BMC neurology.

[261] Korchounov A, Kessler KR, Schipper HI (2004). Differential effects of various treatment combinations on cardiovascular dysfunction in patients with Parkinson's disease. Acta neurologica Scandinavica.

[262] Pitcher TL, Macaskill MR, Anderson TJ (2014). Trends in antiparkinsonian medication use in new zealand: 1995-2011. Parkinson's disease.

[263] Tambasco N, Muti M, Chiarini P, Tarducci R, Caproni S, Castrioto A, Nigro P, Parnetti L, Floridi P, Rossi A, Calabresi P (2014). Entacapone reduces cortical activation in Parkinson's disease with wearing-off: a f-MRI study. PLoS One.

[264] Agundez JA, Garcia-Martin E, Alonso-Navarro H, Jimenez-Jimenez FJ (2013). Anti-Parkinson's disease drugs and pharmacogenetic considerations. Expert opinion on drug metabolism & toxicology.

[265] Marsala SZ, Gioulis M, Ceravolo R, Tinazzi M (2012). A systematic review of catechol-0-methyltransferase inhibitors: efficacy and safety in clinical practice. Clinical neuropharmacology.

[266] Kaakkola S (2010). Problems with the present inhibitors and a relevance of new and improved COMT inhibitors in Parkinson's disease. International review of neurobiology.

[267] Graham DJ, Williams JR, Hsueh YH, Calia K, Levenson M, Pinheiro SP, Macurdy TE, Shih D, Worrall C, Kelman JA (2013). Cardiovascular and mortality risks in Parkinson's disease patients treated with entacapone. Movement disorders: official journal of the Movement Disorder Society.

[268] Ruottinen HM, Rinne UK (1998). COMT inhibition in the treatment of Parkinson's disease. Journal of neurology.

[269] Kaakkola S, Gordin A, Mannisto PT (1994). General properties and clinical possibilities of new selective inhibitors of catechol O-methyltransferase. General pharmacology.

[270] Maltête D, Cottard AM, Mihout B, Costentin J (2011). Erythrocytes catechol-o-methyl transferase activity is up-regulated after a 3-month treatment by entacapone in parkinsonian patients. Clinical neuropharmacology.

[271] Tuomainen P, Reenilä I, Männistö PT (1996). Validation of assay of catechol-O-methyltransferase activity in human erythrocytes. Journal of pharmaceutical and biomedical analysis.

[272] Silindir M, Ozer AY (2014). The benefits of pramipexole selection in the treatment of Parkinson's disease. Neurological sciences: official journal of the Italian Neurological Society and of the Italian Society of Clinical Neurophysiology.

[273] Hauser RA, Schapira AH, Barone P, Mizuno Y, Rascol O, Busse M, Debieuvre C, Fraessdorf M, Poewe W (2014). Long-term safety and sustained efficacy of extended-release pramipexole in early and advanced Parkinson's disease. European journal of neurology: the official journal of the European Federation of Neurological Societies.

[274] Barone P, Schapira AH, Rascol O, Debieuvre C, Fräßdorf M Parkinson's disease.

[275] Mínguez-Mínguez S, Solís-García Del Pozo J, Jordán J (2013). Rasagiline in Parkinson's disease: a review based on meta-analysis of clinical data. Pharmacological research: the official journal of the Italian Pharmacological Society.

[276] Al-Nuaimi SK, Mackenzie EM, Baker GB (2012). Monoamine oxidase inhibitors and neuroprotection: a review. American journal of therapeutics.

[277] Hoy SM, Keating GM (2012). Rasagiline: a review of its use in the treatment of idiopathic Parkinson's disease. Drugs.

[278] Uzbekov MG, Alferova VV, Misionzhnik EY, Gekht AB (2011). Change in platelet monoamine oxidase activity in the acutest period of ischemic stroke is associated with the degree of neurological recovery. Bulletin of experimental biology and medicine.

[279] Carradori S, Secci D, Bolasco A, Chimenti P, D'Ascenzio M (2012). Patent-related survey on new monoamine oxidase inhibitors and their therapeutic potential. Expert opinion on therapeutic patents.

[280] Vilhena RD, Pontes FL, Marson BM, Ribeiro RP, Carvalho KA, Cardoso MA, Pontarolo R (2014). A new HILIC-MS/MS method for the simultaneous analysis of carbidopa, levodopa, and its metabolites in human plasma. Journal of chromatography B, Analytical technologies in the biomedical and life sciences.

[281] Faulkner MA (2014). Safety overview of FDA-approved medications for the treatment of the motor symptoms of Parkinson's disease. Expert opinion on drug safety.

[282] Jost WH (2014). Unwanted effects and interaction of intrajejunal levodopa/carbidopa administration. Expert opinion on drug safety.

[283] Camargo SM, Vuille-Dit-Bille RN, Mariotta L, Ramadan T, Huggel K, Singer D, Goetze O, Verrey F (2014). The molecular mechanism of intestinal levodopa absorption and its possible implications for the treatment of Parkinson's disease. The Journal of pharmacology and experimental therapeutics.

[284] Connolly BS, Lang AE (2014). Pharmacological treatment of Parkinson disease: a review. JAMA: the journal of the American Medical Association.

[285] Bargiotas P, Konitsiotis S (2013). Levodopa-induced dyskinesias in Parkinson's disease: emerging treatments. Neuropsychiatric disease and treatment.

[286] Poletti M, Bonuccelli U (2013). Acute and chronic cognitive effects of levodopa and dopamine agonists on patients with Parkinson's disease: a review. Therapeutic advances in psychopharmacology.

[287] Rodgers KJ, Hume PM, Morris JG, Dean RT (2006). Evidence for L-dopa incorporation into cell proteins in patients treated with levodopa. Journal of neurochemistry.

[288] Salat D, Tolosa E (2013). Levodopa in the treatment of Parkinson's disease: current status and new developments. Journal of Parkinson's disease.

[289] Torsdottir G, Sveinbjornsdottir S, Kristinsson J, Snaedal J, Johannesson T (2006). Ceruloplasmin and superoxide dismutase (SOD1) in Parkinson's disease: a follow-up study. Journal of the neurological sciences.

[290] Kuric E, Ruscher K (2014). Reversal of stroke induced lymphocytopenia by levodopa/benserazide treatment. Journal of neuroimmunology.

[291] Ossig C, Reichmann H (2013). Treatment of Parkinson's disease in the advanced stage. Journal of neural transmission.

[292] Anonymous (2013). Drugs for Parkinson's disease. Treatment guidelines from the Medical Letter.

[293] Rascol O (2013). Extended-release carbidopa-levodopa in Parkinson's disease. Lancet neurology.

[294] Mao Z, Hsu A, Gupta S, Modi NB (2013). Population pharmacodynamics of IPX066: an oral extended-release capsule formulation of carbidopa-levodopa, and immediate-release carbidopa-levodopa in patients with advanced Parkinson's disease. J Clin Pharmacol.

[295] Raz A, Lev N, Orbach-Zinger S, Djaldetti R (2013). Safety of perioperative treatment with intravenous amantadine in patients with Parkinson disease. Clinical neuropharmacology.

[296] Chan HF, Kukkle PL, Merello M, Lim SY, Poon YY, Moro E (2013). Amantadine improves gait in PD patients with STN stimulation. Parkinsonism & related disorders.

[297] Wu FG, Yang P, Zhang C, Li B, Han X, Song M, Chen Z (2014). Molecular Interactions between Amantadine and Model Cell Membranes. Langmuir: the ACS journal of surfaces and colloids.

[298] Reinhart WH, Geissmann-Ott C, Bogdanova A (2011). Activation of N-methyl D-aspartate (NMDA) receptors has no influence on rheological properties of erythrocytes. Clinical hemorheology and microcirculation.

[299] Föller M, Geiger C, Mahmud H, Nicolay J, Lang F (2008). Stimulation of suicidal erythrocyte death by amantadine. European journal of pharmacology.

[300] Herrmann A, Lentzsch P, Lassmann G, Ladhoff AM, Donath E (1985). Spectroscopic characterization of vesicle formation on heated human erythrocytes and the influence of the antiviral agent amantadine. Biochimica et biophysica acta.

[301] Pretorius E (2013). The adaptability of red blood cells. Cardiovascular diabetology.

[302] Lupescu A, Bissinger R, Jilani K, Lang F (2013). Triggering of suicidal erythrocyte death by celecoxib. Toxins.

[303] Flaten TP, Aaseth J, Andersen O, Kontoghiorghes GJ (2012). Iron mobilization using chelation and phlebotomy. Journal of trace elements in medicine and biology: organ of the Society for Minerals and Trace Elements (GMS).

[304] Ma Y, Zhou T, Kong X, Hider RC (2012). Chelating agents for the treatment of systemic iron overload. Curr Med Chem.

[305] Yu Y, Gutierrez E, Kovacevic Z, Saletta F, Obeidy P, Suryo Rahmanto Y, Richardson DR (2012). Iron chelators for the treatment of cancer. Curr Med Chem.

[306] Lu M, Hu G (2012). Targeting metabolic inflammation in Parkinson's disease: implications for prospective therapeutic strategies. Clinical and experimental pharmacology & physiology.

[307] Ton TG, Jain S, Biggs ML, Thacker EL, Strotmeyer ES, Boudreau R, Newman AB, Longstreth WT, Checkoway H (2012). Markers of inflammation in prevalent and incident Parkinson's disease in the Cardiovascular Health Study. Parkinsonism & related disorders.

[308] Patel KV, Ferrucci L, Ershler WB, Longo DL, Guralnik JM (2009). Red blood cell distribution width and the risk of death in middle-aged and older adults. Archives of internal medicine.

[309] Darzynkiewicz Z, Zhao H, Halicka HD, Li J, Lee YS, Hsieh TC, Wu JM (2014). In search of antiaging modalities: evaluation of mTOR- and ROS/DNA damage-signaling by cytometry. Cytometry Part A. the journal of the International Society for Analytical Cytology.

[310] Bové J, Prou D, Perier C, Przedborski S (2005). Toxin-induced models of Parkinson's disease. NeuroRx: the journal of the American Society for Experimental NeuroTherapeutics.

[311] Bonilla-Ramirez L, Jimenez-Del-Rio M, Velez-Pardo C (2011). Acute and chronic metal exposure impairs locomotion activity in Drosophila melanogaster: a model to study Parkinsonism. Biometals: an international journal on the role of metal ions in biology, biochemistry, and medicine.

[312] Du XX, Xu HM, Jiang H, Song N, Wang J, Xie JX (2012). Curcumin protects nigral dopaminergic neurons by iron-chelation in the 6-hydroxydopamine rat model of Parkinson's disease. Neuroscience bulletin.

[313] Febbraro F, Andersen KJ, Sanchez-Guajardo V, Tentillier N, Romero-Ramos M (2013). Chronic intranasal deferoxamine ameliorates motor defects and pathology in the alpha-synuclein rAAV Parkinson's model. Experimental neurology.

[314] Levenson CW, Cutler RG, Ladenheim B, Cadet JL, Hare J, Mattson MP (2004). Role of dietary iron restriction in a mouse model of Parkinson's disease. Experimental neurology.

[315] Xu Q, Kanthasamy AG, Reddy MB (2008). Neuroprotective effect of the natural iron chelator, phytic acid in a cell culture model of Parkinson's disease. Toxicology.

